# Longitudinal Changes in Human Milk Minerals and Vitamins in the Chinese Population: A Scoping Review

**DOI:** 10.3390/nu16111710

**Published:** 2024-05-30

**Authors:** Qiqi Ren, Kaifeng Li, Jufang Li, Jiancun Pan, Yang Liu, Yong Chen, Yajun Xu, Qinggang Xie

**Affiliations:** 1 Heilongjiang Feihe Dairy Co., Ltd., C-16, 10A Jiuxianqiao Rd., Chaoyang, Beijing 100015, China; rqqrenqiqi@163.com (Q.R.); likaifeng@feihe.com (K.L.); lijufang@feihe.com (J.L.); panjiancun@feihe.com (J.P.);; 2 PKUHSC—China Feihe Joint Research Institute of Nutrition and Healthy Lifespan Development, Xueyuan Road 38, Haidian, Beijing 100083, China; 3 Department of Nutrition and Food Hygiene, School of Public Health, Peking University, Xueyuan Road 38, Haidian, Beijing 100083, China; 4 Beijing Key Laboratory of Toxicological Research and Risk Assessment for Food Safety, Peking University, Xueyuan Road 38, Haidian, Beijing 100083, China

**Keywords:** macro mineral elements, trace elements, water-soluble vitamins, fat-soluble vitamins, breast milk, composition, dynamic, profile

## Abstract

This scoping review aims to investigate longitudinal changes in minerals and vitamins concentrations in human milk among the Chinese population. Following the PRISMA-ScR guidelines, a comprehensive and systematic literature search was conducted using both English and Chinese databases. Data were extracted and categorized into six defined lactation stages. We found that the concentration of most minerals decreased throughout the lactation period, although calcium (Ca) and magnesium (Mg) fluctuated slightly across lactation periods. Fat-soluble vitamins also showed a decline throughout the lactation period, while water-soluble vitamins exhibited an increasing trend. However, folic acid, biotin, and pantothenic acid demonstrated a downward trend. Overall, this review has identified the longitudinal changes in minerals and vitamins concentrations in human milk among the Chinese population. In order to conduct a more in-depth examination of maternal characteristics and nutritional factors of the composition of human milk, it is recommended to utilize standardized protocols for the collection and analysis of human milk samples.

## 1. Introduction

Human milk provides all the necessary components for the growth and development of infants, including macronutrients (protein, fat, and carbohydrates), micronutrients (vitamins and minerals), and numerous important bioactive factors [1]. The World Health Organization (WHO) recommends exclusive breastfeeding within the first 6 months of life, with no additional fluid or food needed. It is also advised to continue breastfeeding after 6 months while introducing complementary foods [2]. Nutrients in the human milk and infant body storage accumulated via the placenta during fetal development satisfy the nutritional requirements during early infancy [3]. The investigation into human milk components is currently one of the best ways to understand infant nutrition and health. Human milk constituents change dynamically during lactation. The concentrations of cholesterol and oligosaccharides such as 2′-fucosyllactose decrease over the lactation period [4,5]. The concentrations of amino acids, lactoferrin, and immunoglobulin decrease in the initial stages of lactation [6,7]. By contrast, the levels of total fat and oligosaccharides such as 3′-fucosyllactose increase throughout lactation [4,5]. Presumably, the dynamics surrounding the intake of human milk components indicate the precise needs for nutrients of infants. For instance, concentrations of human milk total amino acids, which are the building blocks of infant body tissue, in 5–6 months of lactation are about two thirds of those available in the first month [6]. This observation coincides with the fact that body weight gain in infants aged 5–6 months is about two thirds of that recorded in infants aged 0–1 month [7].

Minerals and vitamins play a crucial role in the growth, development, and overall health of infants [8]. Numerous studies have examined the levels of these essential nutrients in human milk throughout the lactation period [9,10,11,12]. However, these initial studies often have limitations, such as small sample sizes and a limited number of lactation periods. As a result, the dynamics of these nutrients throughout lactation have not been fully clarified. To address the limitations of individual research articles, systematic review studies have been conducted to compile data from multiple original research studies. However, there are only a few review studies that have specifically investigated the longitudinal changes in minerals and vitamins in human milk. For instance, Dror et al. focused on iodine and vitamin B-12 [13,14], Zhang H et al. examined vitamin A [15], Xi Y et al. reported on vitamin E [16], and Gidrewicz et al. provided data on calcium and phosphorus from 1 to 12 postnatal weeks [17]. Another study by Yang et al. surveyed nine minerals, but they only analyzed milk samples beyond 2 postnatal weeks: by then, the samples contain mature milk. It is crucial to recognize that the composition of human milk remains dynamic even beyond two weeks post birth [18]. Significantly, China has achieved considerable progress in the field of human milk research over recent years, with many of these discoveries published in Chinese. Regrettably, there is an absence of reviews that offer longitudinal data on the mineral and vitamin levels in human milk throughout lactation within the Chinese population.

The existing knowledge gaps obstruct our comprehension of human milk and its critical role in fulfilling the nutritional needs of infants. Consequently, this research aims to bridge these gaps by performing a scoping review to analyze the dynamic changes in minerals and vitamins through different stages of lactation within the Chinese population. In order to accomplish this, a bilingual group compiled and analyzed all available studies published in both Chinese and English. Our efforts will advance the understanding of human milk components and infant nutrition needs.

## 2. Materials and Methods

### 2.1. Literature Search and Selection

This scoping review was conducted following the Preferred Reporting Items for Systematic Reviews and Meta-Analyses guidelines extension for scoping reviews (PRISMA-ScR) [19]. The research question was “What is known about the longitudinal changes in minerals and vitamins in human milk throughout lactation in the Chinese population?”. A comprehensive literature search was conducted using three English databases (PubMed, Web of Science, ScienceDirect) and three Chinese databases (China National Knowledge Infrastructure (CNKI), Chongqing VIP Information, and Wanfang). For English databases, the following search strategy was applied: “(human OR breast) AND (milk) AND (mineral* OR vitamin* OR trace element*) AND (quantification OR concentration* OR content*) AND (China OR Chinese)”. For Chinese databases, we optimized the search strategy to (human milk OR breast milk) AND (mineral OR vitamin OR trace element) per the style of Chinese language. The search concluded in August 2022. Duplicate articles were eliminated, and titles and abstracts of the remaining articles were screened for relevance. The full text of the selected articles was then reviewed for qualification according to the criteria set forth in the PICOS framework (Table 1). Moreover, an assessment of the quality of all the included articles was conducted (Supplementary Table S1). Two investigators, Q.R. and K.L., independently conducted the literature search and selection process. Any discrepancies were resolved with the assistance of a third investigator, Q.X.

### 2.2. Data Extraction and Statistical Analysis

Lactation was categorized into six distinct periods: 1–7, 8–30, 31–60, 61–120, 121–240, and 241–365 postnatal days. Classification of lactation was determined based on the predominant period during which data were gathered. The concentration of elements was summarized in terms of means and standard deviations (SDs). Medians, interquartile rank, and ranges were converted into means as previously described [20]. A density of 103 g/100 mL was applied for the conversion of mg or μg/g of human milk to mg or μg/L [21]. Weighted means, SDs, and standard errors (SEs) of elements in each stage of lactation were calculated. The 95% confidence intervals (CIs) for each element across lactation stages were estimated as weighted mean ± t × SE, where t was calculated from a t-distribution with degrees of freedom equivalent to the sample size of the group minus 1. Cochran’s *Q* statistic from a fixed effect model, where heterogeneity conforms to a *X*^2^—distribution with degrees of freedom equal to the number of subgroups minus 1, was conducted. A random effect model was used when significant heterogeneity was observed using the fixed model. The calculation of Higgins and Thompson’s *I*^2^ was based on the *Q* statistic. Figures were produced with the ggplot2 package in R. The statistical computations were carried out utilizing R software (version 4.0.3). Forest plots were constructed to compare element concentrations from the studies included utilizing Stata/SE 14.0 (StataCorp LLC, College Station, TX, USA). Data mining and statistical analysis were independently conducted by two researchers (Q.R. and K.L.). Any discrepancies were resolved with the involvement of a third researcher (Q.X.).

## 3. Results

**Figure 1:**
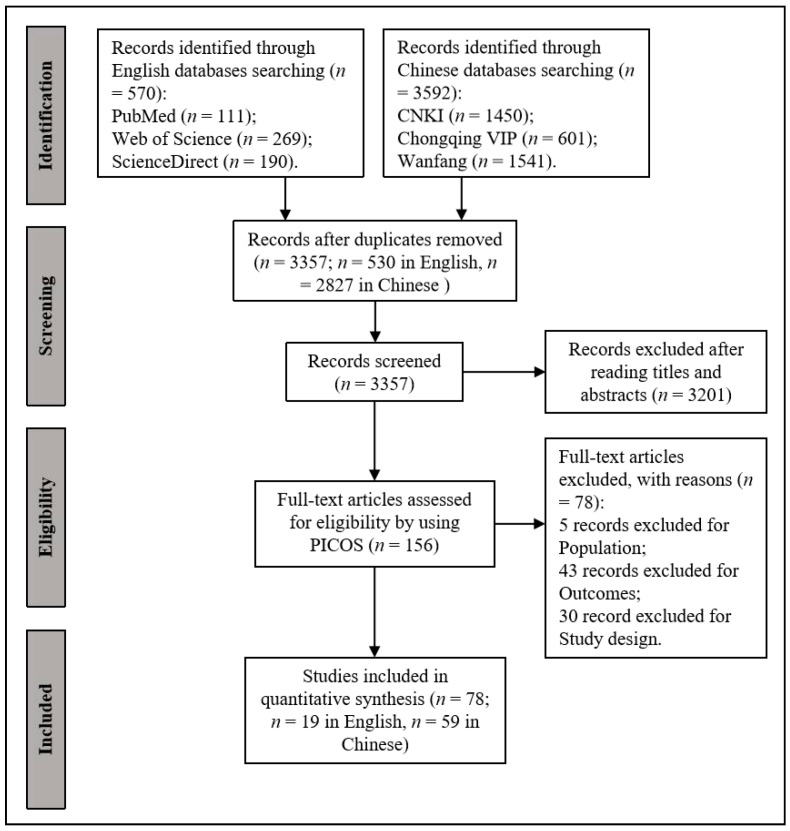
Flow chart of the literature searching and screening process.

**Figure 2:**
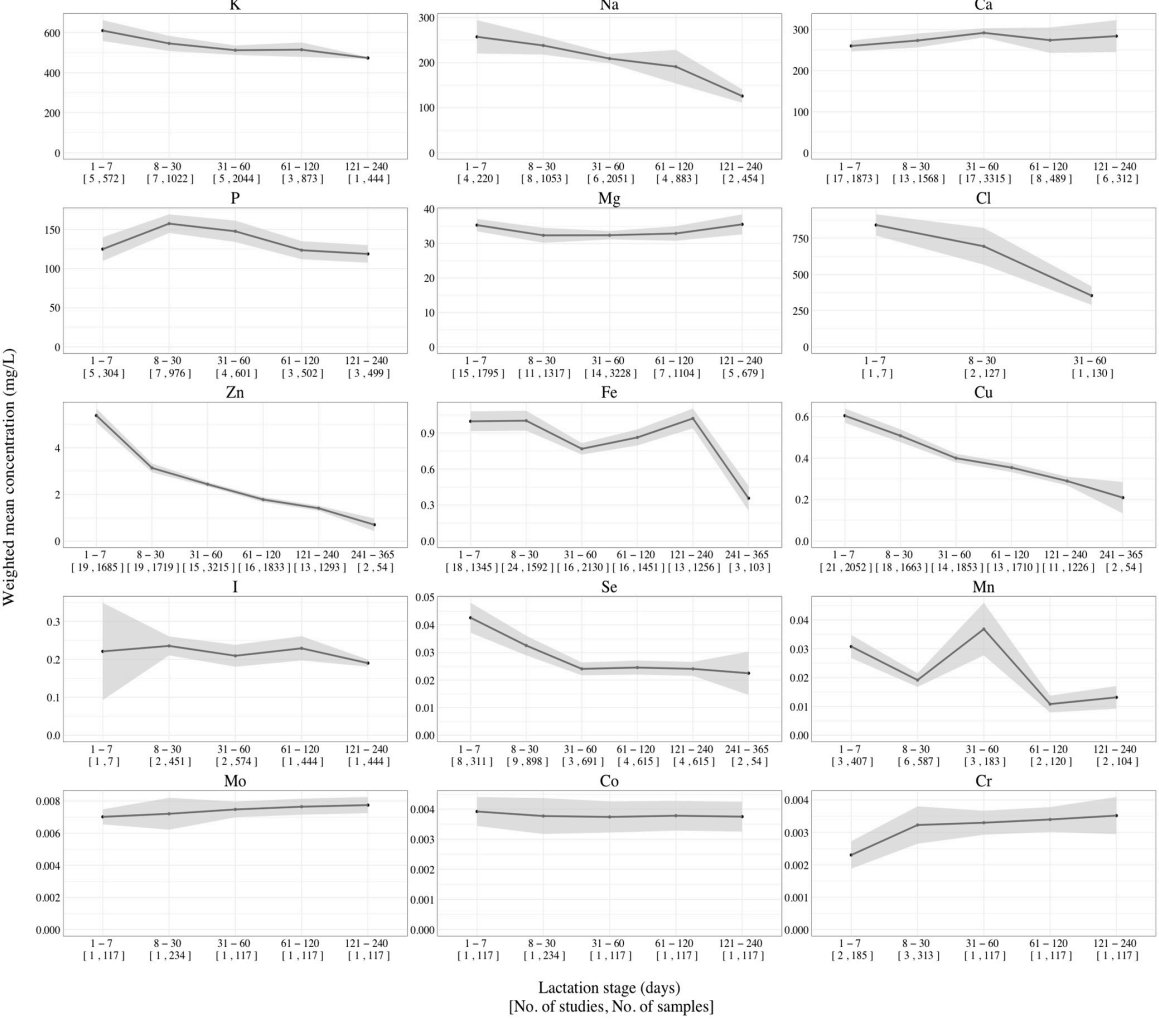
The longitudinal changes in mineral and trace element concentrations in Chinese human milk. Note: shaded areas represent the 95% CI of each feature.

**Figure 3:**
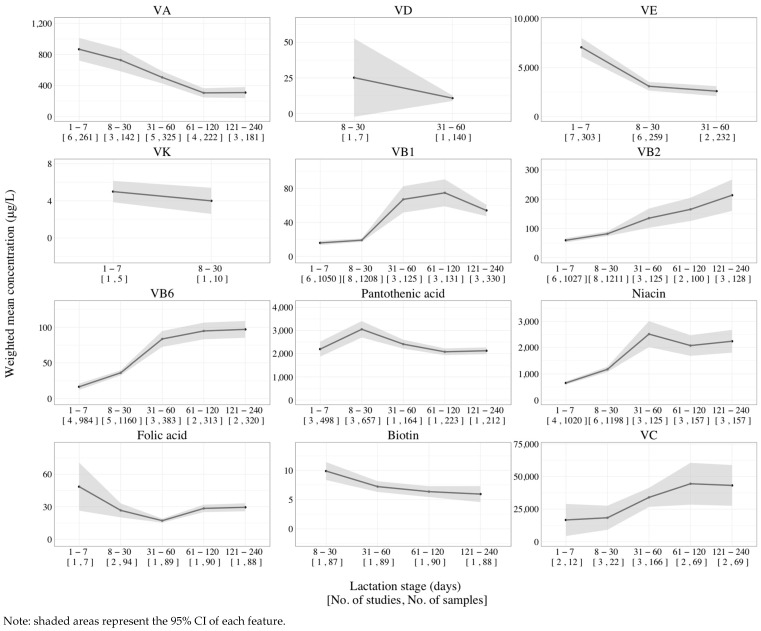
The longitudinal changes in vitamin concentrations in the human milk of the Chinese population.

Figure 1 illustrates the process of literature searching and screening. Eventually, a total of 78 studies were included, comprising 19 in English and 59 in Chinese [10,12,22,23,24,25,26,27,28,29,30,31,32,33,34,35,36,37,38,39,40,41,42,43,44,45,46,47,48,49,50,51,52,53,54,55,56,57,58,59,60,61,62,63,64,65,66,67,68,69,70,71,72,73,74,75,76,77,78,79,80,81,82,83,84,85,86,87,88,89,90,91,92,93,94,95,96,97]. All the included studies are listed in Supplementary Tables S2 and S3. The concentrations of 18 minerals and 12 vitamins were retrieved from these studies, including potassium (K), sodium (Na), calcium (Ca), phosphorus (P), magnesium (Mg), chlorine (Cl), zinc (Zn), iron (Fe), copper (Cu), iodine (I), selenium (Se), manganese (Mn), molybdenum (Mo), cobalt (Co), chromium (Cr), fluorine (F), strontium (Sr), barium (Ba), vitamin A (VA), vitamin D (VD), vitamin E (VE), vitamin K (VK), thiamine (VB1), riboflavin (VB2), vitamin B-6 (VB6), biotin, folic acid, niacin, pantothenic acid, and vitamin C (VC). The forest plots (Supplementary Figures S1–S4) were employed to visualize the heterogeneities present among the included studies. Significant heterogeneities among different studies were observed for both minerals and vitamins. To understand the longitudinal variation of each element during the stages of lactation, a more detailed subgroup heterogeneity analysis was conducted. This analysis employed both fixed effect and random effect models to capture the complexities of the data (Table 2, Figure 2 and Figure 3). The results of fixed effect model analysis suggest significant longitudinal heterogeneities across lactation stages for most of the nutrients studied, except for molybdenum, cobalt, vitamin D, and Vitamin K (Table 2).

Longitudinal changes in the concentration of mineral and trace elements in Chinese human milk are presented in Figure 2, and the results from the subgroup heterogeneity analysis are summarized in Table 2. In general, significant heterogeneities were found for Mg, Cu, Zn, and Fe under the random effect model. The changing patterns of different minerals in Chinese human milk were found to vary throughout lactation. The concentrations of K, Na, Cl, Zn, Fe, Cu, I, Se, and Co decreased over the course of lactation, while Cr and Mo contents increased. Among them, the concentration of Fe fluctuated from 0.8 mg/L to 1.0 mg/L until 240 days postpartum and sharply decreased to 0.4 mg/L thereafter (Supplementary Table S4). Se levels were found to be highest (0.04 mg/L) in the first 7 postnatal days, gradually decreasing thereafter and reaching a plateau (0.02 mg/L) between 31 and 365 postnatal days. The concentrations of Ca and P initially increased during the early stages of lactation and then declined for the rest of lactation, while magnesium showed the opposite trend. The concentrations of Mn showed a significantly higher level during 31–60 postnatal days compared to before and after. It is important to note that only one study was included with respect to the 241–365 days of lactation, resulting in limited data available for this period. On the other hand, limited research has been conducted on the levels of Sr, Ba, and F in human milk among the Chinese population. As a result, the mean values of some of these elements were not extracted during different lactation stages.

Longitudinal changes in the concentration of fat-soluble vitamins and water-soluble vitamins in Chinese human milk are presented in Figure 3, and the results from the subgroup heterogeneity analysis are summarized in Table 2. Longitudinal studies conducted on the fat-soluble vitamins revealed a gradual decrease from colostrum to mature milk. Notably, there was no difference in the VA content between the periods of 61–120 days (306 μg/L) and 121–240 days (310 μg/L) postpartum (Supplementary Table S4). The review observed that the concentrations of VB1, VB2, VB6, niacin, and VC increased over the course of lactation, while folic acid, biotin, and pantothenic acid contents decreased. Among them, the levels of VC eventually stabilized at 43–44 mg/L after 60 days of lactation. Pantothenic acid content showed an initial increase in the first 30 days of lactation, followed by a subsequent decrease. However, there is only one study available on pantothenic acid levels after 30 days of lactation. In addition, there were only two studies available for VD, VK, biotin, and folic acid, and only one study on VE content in human milk after 60 days of lactation.

The ratio of different mineral and vitamin concentrations in human milk may affect the infant absorption and utilization of nutrients as well as the infant health. The ratios of different minerals and vitamins were also calculated, and are shown in Table 3. The potassium to sodium ratio in Chinese human milk remained relatively stable at around 2.5:1 (ranging from 2.3:1 to 2.7:1) across the first 120 days of lactation, then increasing to 3.8:1 from days 121 to 240 postpartum. A similar pattern has been observed for the calcium to phosphorus ratio, which increased from 1.9 during the first 120 days to 2.4 afterwards. The potassium to magnesium ratio in Chinese human milk was found to gradually decrease from 17.6:1 to 13.1:1. On the other hand, during the first 120 days of lactation, the vitamin E to vitamin A ratio in Chinese human milk was found to be 7.3:1 (ranging from 4.3:1 to 8.2:1). From days 121 to 240 of lactation, the ratio was found to increase to 8.2:1.

## 4. Discussion

Longitudinal changes in minerals and vitamins were found to be almost identical to those reported in previous studies conducted in other populations, but there are several differences. Two studies conducted in Japan have also examined the changes in mineral and trace element composition in human milk over the lactation period [98,99]. Specifically, Na, K, Cr, and Zn were found to demonstrate a decreasing trend. Although iron also showed a downward trend, the change was not statistically significant. The trends of Mg and Ca were found to fluctuate slightly throughout lactation. However, our results suggest that there is no significant decline in Cr during lactation, a finding which may be influenced by the fact that only one article on chromium concentration covering the whole lactation period was included in this study. A study by Sakurai T et al. [100] demonstrated that the mean concentrations of VA, VE, and VD in Japanese mothers exhibited a decreasing trend during lactation. Similarly, a study conducted on the Brazilian population by Campos JM et al. [101] yielded similar findings. Another study by Ford et al. [102] revealed that the mean concentrations of VB1, VB6, niacin, pantothenic acid, biotin, and folic acid in British mothers increased progressively with the stage of lactation. This trend was also observed in a study conducted on the Japanese population by Sakurai T et al. [100]. However, our findings regarding VC in human milk are inconsistent with those reported in non-Chinese populations. Ahmed L Jr et al. [103] demonstrated that the concentration of vitamin C in human milk from mothers in Bangladesh is at its peak in colostrum and decreases as lactation progresses (3.52 ± 0.56 mg/dL in colostrum compared to 3.03 ± 0.67 mg/dL in mature milk). A normal distribution is usually extensively and implicitly assumed in meta-analyses [104], a fact which is important to consider when interpreting the statistics of this review. This is typically true at the between-study level for random effects models where the central limit theorem is not guaranteed for a large sample size [105]. In our study, data for VA, VE, VB1, VB2, VC, Na, Zn, Fe, Cu, Se, Mn, Co, and Cr seem to be not normally distributed (*p* < 0.05) according to the Shapiro–Wilk test (Supplementary Table S5); therefore, the random effects of those features should be interpreted with caution.

Human milk offers an invaluable source of energy and nutrients critical for the growth and development of infants younger than six months. Its composition is uniquely tailored to meet the specific nutritional requirements of each individual infant. Nonetheless, there are circumstances where exclusive breastfeeding may not be feasible. In such situations, infant formula becomes an indispensable alternative, providing the essential nutrients required for infant growth and development when breastfeeding is not an option. Typically, infant formula is formulated based on dietary reference intakes (DRIs). In this study, we estimated the daily average lactation volume of Chinese lactating mothers within 6 months after delivery to be 750 mL. The nutrient content data collected in this review for each lactation period were converted to obtain the daily intake and compared with the Chinese Dietary Reference Intakes and the American Dietary Reference Intakes [106,107] (Supplementary Table S6). These findings can offer valuable insights for the development of infant formula in China. Longitudinal changes in nutrient content in human milk can serve as reference values for infant needs. However, special circumstances may arise, such as the low vitamin D content in human milk which makes it difficult to meet the recommended intake of 400 IU daily. Human milk and sun exposure constitute the natural sources of vitamin D for an infant during the first months of life. However, it is currently recommended that infants under six months of age should not be exposed to direct sunlight as the most appropriate photoprotection measure to reduce the risks of skin cancer. Therefore, infants need pharmacological vitamin D supplementation [108]. Vitamin K, on the other hand, is obtained from human milk and synthesized by the baby’s own intestinal microbes. In general, not all the nutritional needs of infants can be fully based on human milk. Nevertheless, it remains challenging to determine the relative influence of different sources of vitamin D and vitamin K on the nutritional status of infants and young children.

The temporal trajectories of the concentration of minerals and vitamins in human milk are important to guide the exploration of infant nutritional needs. The proportion of nutrients is also critical, as different ratios may affect the absorption and utilization of nutrients in infants. This study reviewed some important mineral and vitamin ratios. Na and K are essential nutrients that play crucial roles in various physiological functions, such as the maintenance of plasma volume, osmolality, and resting membrane potential [109,110]. Despite their opposing functions, Na and K are closely linked to blood pressure, kidney function, and cardiovascular health [111]. While the topics of dietary sodium and potassium intake, as well as the potassium-to-sodium ratio, are extensively discussed in adult health contexts, there is limited information available regarding infants [112,113,114]. It is noteworthy that nutritional intake during infancy can significantly influence future growth and health outcomes. Human milk is recognized as a natural dietary source for infants, underscoring the importance of understanding the potassium-to-sodium ratio during each lactation period. Our study in China sheds light on the unique potassium-to-sodium ratio in human milk, highlighting the need for further research into its impact on the long-term development of infants. Mg and K play crucial roles in regulating muscle contraction, relaxation, and maintaining myocardial function [115]. Mg serves as an activator for various enzymes and participates in enzymatic reactions as a cofactor. Deficiency in Mg is often linked with hypokalemia, as it can deactivate (Na^+^ + K^+^)-ATPase in renal tubular epithelial cells, leading to impaired potassium reabsorption and excessive potassium loss [116,117]. Our study in China explored the magnesium-to-potassium ratio in human milk. Ca, Mg, and P play a critical role in maintaining bone tissue balance. Any fluctuation in the concentration of these elements can impact each other’s functions. The balance of calcium and phosphorus in the diet is essential for healthy bone development, ideally ranging from a ratio of 1:1 to 2:1. Additionally, Mg acts as a counterbalance to Ca. Elevated Mg levels can alter the Ca/Mg ratio, potentially disrupting cellular processes [118]. Our study uncovered the proportions of calcium, phosphorus, and magnesium in human milk within the Chinese population. This finding provides a broader understanding of the appropriate nutritional requirements to support skeletal development in infants. Complex interactions exist with respect to fat-soluble vitamin absorption at the intestine level, suggesting that the vitamin E may prevent vitamin A oxidation, therefore increasing its intestinal absorption [119]. Our study revealed the ratio of vitamin A to vitamin E in Chinese human milk. Further studies are needed to fully understand the potential impact of this ratio on the absorption of VA and VE.

In addition to the lactation stage, various factors affect the composition of human milk. These factors can be categorized into different aspects. One aspect is individual maternal factors, such as genetic background, maternal health, maternal age, maternal BMI, race, nutritional status, preterm birth, parity, delivery mode, and smoking. For example, the calcium levels in human milk are influenced by the genotype of the vitamin D receptor, with higher levels observed in milk of the bb genotype compared to the aa and tt genotypes [120]. Maternal familial hypophosphatemia or hyperparathyroidism can significantly decrease human milk phosphorus concentrations [121,122]. Women with iron deficiency anemia generally exhibit lower concentrations of calcium in their human milk [123]. However, it is important to note that the mothers considered in this review did not have such diseases. The influence of maternal age on human milk minerals and vitamins concentration is still a topic of debate. Studies have shown that lactating adolescents have lower concentrations of calcium, magnesium, vitamin A, and vitamin E in their human milk [124,125]. Research conducted in low-income areas has found an inverse relationship between maternal age and the content of iron, zinc, and copper in human milk [126]. However, some studies suggest that maternal age does not affect human milk calcium, copper, and vitamin A concentrations [127,128,129,130]. It is important to note that this review did not include any adolescent mothers. Previous studies have uncovered a positive correlation between the copper content in human milk and the BMI of lactating mothers. Additionally, there is a correlation between human milk iodine concentration and the body weight of lactating mothers [131,132]. A study has shown that race affects vitamin D levels in human milk, with black people exhibiting lower levels than white people [133]. The mothers included in this review are all Chinese. However, it is important to note that China is a large country with abundant resources and diverse ethnic groups [134]. In a study conducted by Chen HH et al., included in our review, it was found that the levels of Ca, Mn, Cu, and Zn in Dong’s human milk were significantly lower compared to Han’s human milk [54]. Several research investigations have identified a notable relationship between the selenium levels in serum or plasma and those in human milk [135], although some investigations did not observe this correlation [136]. Other studies have found that human milk vitamin A and vitamin D3 concentrations are closely related to the liver reserve and serum concentration of lactating mothers [137,138,139]. In addition, human milk from preterm mothers has been shown to have slightly higher levels of potassium, copper, iron, zinc, selenium, vitamin C, pantothenic acid, and vitamin B-12 [102,140,141,142], as well as slightly lower levels of vitamins A, D, E, B1, and B6 than human milk from full-term mothers [102,142,143,144,145]. Lower concentrations of zinc and higher concentrations of vitamins A have been observed in human milk of multipara [38,146]. The copper content in human milk has been found to be positively correlated with parity [131]. However, some studies have reported that parity does not influence human milk copper concentrations [129,130]. One investigation revealed an inverse correlation between maternal parity and selenium levels in human milk during late lactation [38], even though this association was not detected in other research [146]. Other studies have shown that the iodine content in the milk of mothers who delivered via cesarean between 5 and 11 days postpartum is significantly higher than that of mothers who had a natural delivery [38]. Studies have found a negative correlation between maternal smoking, on one hand, and iodine and vitamin E levels, on the other, in human milk [147,148]. This review examined the inclusion criteria of the studies, which considered factors such as the gestational status (full-term or preterm), mode of delivery, parity (primiparae or multiparae), type of pregnancy (monocyesis or multiple gestation), and smoking status of the mothers. However, it was noted that most studies did not provide specific details on these factors, making it impossible for us to gather data and examine the association among these characteristics and the levels of minerals and vitamins in human milk. This is a limitation of this review.

The second aspect to consider is dietary factors, including dietary intake, dietary supplements, geographical regions, social and economic conditions, etc. The levels of iodine, selenium, mercury, vitamin A, and vitamin D in human milk have been found to be positively correlated with the intake of these as part of the lactating maternal diet [127,128,146,149]. Similarly, maternal selenium, iodine, vitamin A, vitamin E, vitamin K, and water-soluble vitamins supplementation have been found to effectively increase their concentrations in human milk [10,150,151,152,153,154,155]. The dietary habits and intake of local people may be influenced by the availability of resources in different geographical areas. In regions where there is a low habitual consumption of calcium, the amount of dietary calcium ingested may affect the concentrations of calcium in human milk [156]. Additionally, in locations with elevated levels of algae and seaweed, the levels of iodine in human milk has been shown to be elevated when compared to other regions [157]. Likewise, in areas with a high selenium content in the soil, the maternal intake of organic selenium in the diet has been found to be enhanced, resulting in higher concentrations of selenium in human milk [158]. In addition, a study has shown that low-income nursing mothers in developing countries have lower levels of vitamin A in their milk than human milk from developed countries [159]. The majority of studies included in this review did not focus on the dietary situation of the mothers or provide relevant dietary survey data. This is also a limitation of this review. However, as each study primarily recruited volunteer mothers who resided in the same city and provided human milk, the dietary patterns of mothers living in the same area for an extended period were generally similar. Thus, in cases where a large sample size was used, the impact of individual dietary conditions on the statistical results of longitudinal changes in human milk within the entire group was minimal.

The third aspect to consider is methodological considerations, such as sample collection and detection methods. Research has indicated that the season exerts a considerable influence on the concentration of vitamin D in human milk. Specifically, elevated levels of vitamin D have been observed during the summer and autumn when compared to the winter and spring. Furthermore, studies have demonstrated that the quantities of iron and folate in human milk are higher when the milk is collected in the afternoon or evening as opposed to the morning [129,160]. Furthermore, research has found that hind-milk had higher concentrations of iron, selenium, and vitamin D [129,161]. It is worth noting that almost all of the studies included in this review collected human milk in the morning using a breast pump for full expression. As for the detection methods, the most commonly used method for measuring K, Na, Ca, Mg, Zn, Fe, Cu, and Mn was AAS, followed by ICP-MS and ICP-AES. Ammonium date colorimetry was commonly used for testing P, while AFS was the common method for testing Se. Lastly, HPLC was the most frequently employed method for vitamin detection.

## 5. Conclusions

This research combined data on the levels of minerals and vitamins found in Chinese human milk over time to create a comprehensive overview of relevant literature in Chinese and English. Our scoping review indicate that, in general, most mineral levels decrease throughout lactation. Fat-soluble vitamins also decrease over the lactation period, while water-soluble vitamins increase. The mineral and vitamin levels in the human milk of the Chinese population are similar to those found in non-Chinese populations. These data can offer valuable insights for the requirements and actual intakes by infants in China. Additionally, factors other than lactation can affect the concentration of minerals and vitamins in human milk. Therefore, we also discuss the impact of individual maternal factors, dietary factors, and methodological considerations. However, a common flaw in most studies is the lack of specific details on maternal and dietary factors, making it impossible to extract and analyze the association between these characteristics and the concentration of minerals and vitamins in human milk. Considering the limited available evidence, it is recommended to use standardized procedures for collecting and analyzing human milk samples to delve deeper into the influence of maternal characteristics and dietary factors on human milk composition.

## Figures and Tables

**Table 1:** Inclusion and exclusion criteria for selecting articles (PICOS).

Parameter	Inclusion Criteria	Exclusion Criteria
Population	Chinese population; healthy mothers with healthy neonates.	Non-Chinese populations; non-human; mothers or infants with defined diseases or disorders (premature delivery was not regarded as a disease or disorder).
Intervention	N/A	N/A
Comparator	N/A	N/A
Outcomes	Human milk samples; data were expressed as means or medians; lactation stages could fit into the categories of 1–7 d, 8–30 d, 31–60 d, 61–120 d, 121–240 d, 241–365 postnatal days.	Lactation stage not specified or simply described as colostrum, transition milk, or mature milk.
Study design	Original articles from peer-reviewed journals; master theses or doctoral dissertations that reported original research data.	Review articles; abstracts; articles without access to the full text; milk samples were pooled together before assessment.

Note: N/A, not applicable.

**Table 2:** Results of subgroup heterogeneity analysis.

	Fixed Model				Random Model					Fixed Model				Random Model			
Mineral	*Q*	*df*	*p*	*I* ^2^	*Q*	*df*	*p*	*I* ^2^	*Vitamin*	*Q*	*df*	*p*	*I* ^2^	*Q*	*df*	*p*	*I* ^2^
K	1171.1	4	2.94 × 10^−252^	99.7	3.306	4	0.508	0.0	VA	2797	4	0.000	99.9	5.99	4	0.200	33.2
Na	279.7	4	2.57 × 10^−59^	98.6	0.505	4	0.973	0.0	VD	1.5	1	0.217	34.5	0.2	1	0.662	0.0
Ca	2245.4	4	0.000	99.8	2.365	4	0.669	0.0	VE	624.1	2	3.00 × 10^−136^	100.0	3.9	2	0.142	48.8
P	1166.0	4	3.69 × 10^−251^	99.7	9.370	4	0.052	57.3	VK	1.7	1	0.197	40.0	0.2	1	0.655	0.0
Mg	17,387.1	4	0.000	100.0	23.314	4	1.10 × 10^−4^	82.8	VB1	1154.7	4	1.05 × 10^−248^	99.7	2.1	4	0.721	0.0
Cl	127.9	2	1.70 × 10^−28^	98.4	1.776	2	0.411	0.0	VB2	450.5	4	3.35 × 10^−96^	99.1	0.5	4	0.976	0.0
Zn	4,465,555.1	5	0.000	100.0	156.307	5	6.06 × 10^−32^	96.8	VB6	1087.7	4	3.58 × 10^−234^	99.6	7.5	4	0.113	46.5
Fe	17,461.3	5	0.000	100.0	21.334	5	7.00 × 10^−4^	76.6	Pantothenic acid	17.5	4	0.002	77.1	0.1	4	0.999	0.0
Cu	13,396.2	5	0.000	100.0	211.469	5	9.97 × 10^−44^	97.6	Niacin	699.0	4	5.74 × 10^−150^	99.4	2.2	4	0.691	0.0
I	59.0	4	4.67 × 10^−12^	93.2	0.549	4	0.969	0.0	Folic acid	69.8	4	2.45 × 10^−14^	94.3	1.9	4	0.756	0.0
Se	264.7	5	3.91 × 10^−55^	98.1	1.653	5	0.895	0.0	Biotin	18.4	3	3.64 × 10^−4^	83.7	0.2	3	0.976	0.0
Mn	1191.6	4	1.04 × 10^−256^	99.7	6.105	4	0.191	34.5	VC	78.5	4	3.62 × 10^−16^	94.9	2.7	4	0.615	0.0
Mo	5.6	4	0.228	29.0	0.047	4	1.000	0.0									
Co	0.4	4	0.986	0.0	0.003	4	1.000	0.0									
Cr	313.9	4	1.11 × 10^−66^	98.7	2.958	4	0.565	0.0									

Means are presented as weighted means calculated from the included studies. *Q*: Cochran’s *Q* statistic which follows a chi-square distribution with *df* degrees of freedom; *df*: degrees of freedom; *I*^2^: Higgins and Thompson’s *I*^2^.

**Table 3:** Ratios of different minerals and vitamins.

	1–7 Days	8–30 Days	31–60 Days	61–120 Days	1–120 Days	121–240 Days
K/Na	2.4	2.3	2.4	2.7	2.5	3.8
Ca/P	2.1	1.7	2	2.2	1.9	2.4
Ca/Mg	7.5	8.5	9.1	8.2	8.5	7.9
K/Mg	17.6	17.1	16	15.4	16.2	13.1
P/Mg	3.6	4.9	4.6	3.7	4.4	3.3
VE/VA	8.2	4.3	5.2	8.1	7.3	8.2

## Data Availability

The data presented in this study are available on request from the corresponding author due to privacy reasons.

## Supplementary Materials

The following supporting information can be downloaded at: https://www.mdpi.com/article/10.3390/nu16111710/s1, Table S1: Quality assessment of the included studies; Table S2: List of all included studies for minerals and trace elements; Table S3: List of all included studies for vitamins; Table S4: Longitudinal changes in mineral and vitamin concentrations in human milk in Chinese population; Table S5: Shapiro-Wilk test; Table S6. Comparison of recommended dietary intakes; Figure S1: Forest plot of comparison of macro mineral elements concentrations; Figure S2: Forest plot of comparison of trace elements concentrations; Figure S3: Forest plot of comparison of fat-soluble vitamins concentrations; Figure S4: Forest plot of comparison of water-soluble vitamins concentrations.
