# Supplementary material for: Longitudinal Changes in Human Milk Minerals and Vitamins in the Chinese Population: A Scoping Review
Source: Nutrients. 2024 May 30;16(11):1710. doi: 10.3390/nu16111710 (PMC11174910; doi:10.3390/nu16111710)
Supplement: Supplementary file 1 [file nutrients-16-01710-s001.zip › nutrients-2993327-supplementary.pdf]

Title: Longitudinal Changes in Human Milk Minerals and Vitamins in Chinese Population: A Scoping Review

Supplementary Materials

Supplementary Table S1. Quality assessment of the included studies.

References	Q1	Q2	Q3	Q4	Q5	Q6	Q7	Q8	Q9	Q10	Q11	Q12	Q13	Q14	Q15	Q16	Q17
[1]	Y	NR	Y	Y	Y	Y	NR	Y	Y	Y	Y	Y	NR	Y	Y	NR	CD
[2]	Y	NR	Y	Y	Y	CD	NR	Y	Y	Y	Y	Y	CD	NR	Y	NR	NR
[3]	Y	NR	Y	Y	Y	CD	NR	Y	Y	Y	Y	Y	NR	NR	Y	Y	NR
[4]	NR	NR	Y	Y	Y	Y	NR	Y	NR	NR	NR	Y	Y	NR	Y	NR	Y
[5]	Y	NR	Y	Y	Y	CD	NR	Y	NR	NR	NR	Y	NR	Y	Y	NR	NR
[6]	Y	NR	Y	Y	Y	CD	NR	Y	Y	Y	Y	Y	NR	NR	NR	NR	NR
[7]	NR	NR	Y	Y	Y	CD	NR	Y	NR	Y	NR	Y	Y	NR	Y	NR	NR
[8]	Y	Y	Y	Y	Y	Y	CD	Y	NR	NR	NR	Y	Y	NR	Y	NR	NR
[9]	NR	NR	Y	Y	Y	CD	NR	Y	NR	NR	NR	Y	NR	NR	NR	NR	NR
[10]	Y	Y	Y	Y	Y	Y	NR	Y	NR	NR	NR	Y	NR	Y	Y	NR	NR
[11]	NR	NR	Y	Y	Y	Y	NR	Y	NR	NR	Y	Y	CD	Y	NR	NR	NR
[12]	Y	NR	Y	Y	Y	Y	NR	Y	Y	Y	Y	Y	Y	NR	Y	NR	NR
[13]	Y	Y	Y	Y	Y	Y	NR	Y	NR	NR	CD	Y	NR	NR	NR	NR	NR
[14]	Y	NR	Y	Y	Y	CD	NR	Y	NR	NR	NR	Y	Y	NR	NR	NR	NR
[15]	NR	NR	Y	Y	Y	CD	NR	Y	NR	NR	NR	Y	NR	NR	NR	NR	NR
[16]	Y	NR	Y	Y	Y	CD	NR	Y	NR	NR	NR	Y	NR	NR	NR	NR	NR
[17]	NR	NR	Y	Y	Y	CD	NR	Y	NR	Y	NR	Y	Y	NR	Y	NR	NR

[18]	Y	NR	Y	Y	Y	Y	NR	Y	NR	Y	NR	Y	Y	NR	Y	Y	NR
[19]	NR	NR	Y	Y	Y	Y	NR	Y	Y	Y	Y	Y	Y	NR	NR	Y	NR
[20]	NR	NR	Y	Y	Y	CD	NR	Y	Y	Y	Y	Y	NR	NR	Y	Y	Y
[21]	Y	NR	Y	Y	Y	CD	NR	Y	NR	NR	NR	Y	Y	NR	Y	NR	NR
[22]	Y	NR	Y	Y	Y	CD	NR	Y	NR	NR	Y	Y	NR	NR	Y	NR	NR
[23]	Y	NR	Y	Y	Y	CD	NR	Y	Y	Y	Y	Y	Y	NR	Y	NR	NR
[24]	Y	Y	Y	Y	Y	CD	NR	Y	Y	Y	Y	Y	NR	NR	Y	Y	Y
[25]	NR	NR	Y	Y	Y	CD	NR	Y	NR	NR	NR	Y	NR	NR	NR	NR	NR
[26]	NR	NR	Y	Y	Y	CD	NR	Y	Y	NR	NR	Y	NR	NR	Y	NR	NR
[27]	Y	NR	Y	Y	Y	Y	NR	Y	Y	Y	Y	Y	NR	NR	Y	Y	NR
[28]	Y	NR	Y	Y	Y	CD	NR	Y	NR	NR	NR	Y	NR	NR	Y	Y	NR
[29]	NR	NR	Y	Y	Y	CD	NR	Y	NR	Y	NR	Y	NR	NR	Y	NR	NR
[30]	NR	Y	Y	Y	Y	Y	NR	Y	NR	NR	NR	Y	NR	NR	Y	NR	NR
[31]	Y	NR	Y	Y	Y	CD	NR	Y	NR	NR	NR	Y	NR	Y	NR	NR	Y
[32]	NR	NR	Y	Y	Y	CD	CD	Y	NR	NR	NR	Y	NR	NR	NR	NR	NR
[33]	Y	Y	Y	Y	Y	CD	NR	Y	NR	NR	NR	Y	CD	NR	Y	NR	NR
[34]	Y	NR	Y	Y	Y	CD	NR	Y	NR	Y	NR	Y	NR	NR	Y	NR	NR
[35]	Y	Y	Y	Y	Y	CD	NR	Y	NR	NR	NR	Y	Y94%	NR	Y	NR	NR
[36]	NR	NR	Y	Y	Y	CD	NR	Y	Y	Y	Y	Y	NR	NR	Y	NR	NR
[37]	NR	NR	Y	NR	Y	CD	NR	Y	NR	NR	NR	Y	NR	NR	NR	NR	NR
[38]	NR	Y	Y	Y	Y	CD	NR	Y	NR	NR	Y	Y	CD	NR	Y	NR	NR
[39]	NR	NR	Y	Y	Y	CD	NR	Y	NR	Y	NR	Y	NR	NR	Y	NR	NR
[40]	NR	NR	Y	Y	Y	CD	NR	Y	NR	NR	NR	Y	NR	NR	NR	NR	NR
[41]	Y	Y	Y	Y	Y	CD	NR	Y	NR	Y	NR	Y	NR	NR	Y	NR	NR

[42]	NR	NR	Y	Y	Y	CD	NR	Y	NR	NR	NR	Y	NR	NR	Y	NR	Y
[43]	NR	Y	Y	Y	Y	CD	NR	Y	NR	NR	NR	Y	Y	NR	Y	NR	NR
[44]	Y	Y	Y	NR	CD	CD	NR	Y	NR	NR	NR	Y	NR	NR	Y	NR	NR
[45]	Y	Y	Y	Y	Y	CD	NR	Y	NR	Y	CD	Y	NR	NR	Y	NR	NR
[46]	NR	NR	Y	NR	Y	CD	NR	Y	NR	NR	NR	Y	NR	NR	NR	NR	NR
[47]	NR	NR	Y	NR	Y	CD	NR	Y	NR	NR	Y	Y	NR	NR	Y	NR	NR
[48]	Y	NR	Y	Y	Y	CD	NR	Y	NR	NR	NR	Y	NR	NR	Y	NR	NR
[49]	Y	NR	Y	Y	Y	CD	NR	Y	NR	NR	NR	Y	Y	Y	Y	NR	NR
[50]	Y	NR	Y	Y	Y	CD	NR	Y	NR	Y	NR	Y	Y	NR	Y	NR	NR
[51]	NR	NR	Y	NR	Y	CD	NR	Y	NR	NR	NR	Y	NR	NR	Y	NR	NR
[52]	NR	NR	Y	Y	Y	CD	CD	Y	NR	NR	NR	Y	NR	NR	NR	NR	NR
[53]	NR	NR	Y	NR	Y	CD	NR	Y	NR	NR	NR	Y	NR	NR	NR	NR	NR
[54]	NR	Y	Y	Y	Y	CD	Y	Y	NR	NR	NR	Y	CD	NR	Y	NR	NR
[55]	Y	Y	Y	Y	Y	CD	NR	Y	NR	Y	Y	Y	NR	NR	Y	NR	NR
[56]	Y	NR	Y	Y	Y	CD	NR	Y	NR	NR	NR	Y	CD	Y	Y	NR	NR
[57]	Y	NR	Y	Y	Y	Y	NR	Y	NR	Y	NR	Y	Y	Y	Y	NR	NR
[58]	Y	NR	Y	NR	Y	CD	CD	Y	NR	NR	Y	Y	NR	NR	NR	Y	NR
[59]	NR	NR	Y	Y	Y	CD	Y	Y	NR	NR	NR	Y	NR	NR	NR	Y	NR
[60]	NR	NR	Y	Y	Y	CD	Y	Y	NR	NR	NR	Y	Y	NR	NR	Y	NR
[61]	Y	NR	Y	Y	Y	NR	Y	Y	Y	Y	NR	Y	NR	Y	Y	Y	Y
[62]	Y	NR	Y	CD	Y	NR	Y	Y	Y	Y	NR	Y	NR	Y	Y	Y	NR
[63]	Y	NR	Y	Y	Y	NR	Y	Y	Y	Y	NR	Y	NR	Y	Y	Y	Y
[64]	Y	NR	Y	Y	Y	NR	Y	Y	Y	Y	NR	Y	NR	Y	Y	Y	NR
[65]	Y	NR	Y	Y	Y	NR	Y	Y	Y	Y	NR	Y	NR	Y	Y	Y	NR

[66]	Y	NR	Y	Y	Y	NR	NR	Y	Y	Y	NR	Y	NR	Y	Y	NR	NR
[67]	NR	NR	Y	Y	Y	NR	NR	Y	NR	NR	NR	Y	NR	NR	NR	NR	NR
[68]	Y	NR	Y	Y	Y	NR	NR	Y	NR	NR	NR	Y	NR	NR	NR	NR	NR
[69]	Y	NR	Y	Y	Y	NR	NR	Y	NR	NR	NR	Y	NR	NR	Y	NR	Y
[70]	Y	NR	Y	Y	Y	NR	NR	Y	NR	NR	NR	Y	NR	NR	NR	NR	NR
[71]	NR	NR	Y	Y	Y	NR	N	Y	NR	NR	NR	Y	NR	NR	NR	NR	NR
[72]	Y	NR	Y	Y	Y	NR	NR	Y	Y	Y	NR	Y	NR	Y	Y	Y	NR
[73]	NR	NR	Y	Y	Y	NR	N	Y	Y	Y	NR	Y	NR	NR	Y	Y	NR
[74]	Y	NR	Y	Y	Y	NR	Y	Y	Y	Y	NR	Y	NR	NR	Y	Y	NR
[75]	Y	NR	Y	CD	NR	NR	NR	Y	NR	NR	NR	Y	NR	Y	Y	Y	NR
[76]	Y	NR	Y	Y	Y	NR	NR	Y	NR	NR	NR	Y	NR	NR	NR	NR	NR
[77]	Y	NR	Y	Y	Y	NR	NR	Y	Y	NR	NR	Y	NR	Y	Y	NR	NR
[78]	Y	NR	Y	Y	Y	NR	Y	Y	NR	NR	NR	Y	NR	Y	Y	NR	NR

Note:

Q1: Did the authors clearly describe that the infants were Y or pre-term?

Q2: Did the authors describe whether births were via vaginal delivery or C-section?

Q3: Did the authors clearly describe that the mothers were healthy?

Q4: Did the authors clearly describe the number of mothers recruited in the study?

Q5: Did the authors clearly describe the geographic locations of human milk sample collection?

Q6:Did the authors clearly describe whether the human samples were collected from Urban or rural areas?

Q7: Did the authors study the dietary intakes and supplement of lactating mothers**?**

Q8: Did the authors clearly describe the lactation stage without using the terms colostrum, transition milk, or mature milk?

Q9: Did the authors clearly describe the Fore-milk, hind-milk, full expression, or pooled full expression within 3-4 hours?

Q10: Did the authors clearly describe the time of day when the human milk was collected or whether all the human milk was collected at the same time of the day?

Q11: Did the authors describe whether the human milk was collected via manual operation or breast pump?
Q12: Did the authors provide detailed methods for measuring the minerals and vitamins concentration?
Q13: Did the authors clearly describe the primipara or not?
Q14: Did the authors clearly describe the single or multiple births?
Q15: Did the authors clearly describe the maternal age?
Q16: Did the authors clearly describe the mother smokes or not?
Q17: Did the authors clearly describe the maternal ethnicity?
CD, cannot be determined; NR, not reported; N, no; Y, yes.

Supplementary Table S2. List of all included studies for minerals and trace elements

References	City	Reported outcomes	Quantification Methods	Lactation (d)	Numbers of mothers	Numbers of samples	Full-term or pre-term	Fore-milk or hind-milk or full expression	Collection time	Manual or pump	Ethnic	Dietary supplements intake	Urban or rural	Maternal age (years of age)	Delivery	Primipara or multipara	Monocyesis or multiple gestation	Smoking or not
[1]	Beijing<br>Guangzhou, Guangdong<br>Suzhou, Jiangsu	Fe, Zn, Cu, Ca, P, K, Na, Mg, Se, I	ICP-MS	5-11<br>12-30<br>31-60<br>61-120<br>121-240	444	444<br>444<br>444<br>444<br>444	Full-term	Full expression	9-11 a.m	Pump	Han (95.6%)	N.S.	Urban	18-45	N.S.	N.S.	Monocyesis	N.S.
[2]	Inner Mongolia	Ca, Mg, Cu, Fe, Mn, Na, K, Zn;<br>P;<br>Cl;<br>I;<br>Se	AAS;<br>Spectrophotometer;<br>Potentiometric Titration;<br>HPLC;<br>AFS	3-7<br>8-21<br>22-180	66	7<br>7<br>66	Full-term	Full expression	9-11 a.m	Pump	N.S.	N.S.	N.S.	28.3±3.9	N.S.	Primipara (83.3%)	N.S.	N.S.
[3]	Qiqihar, Heilongjiang	Fe, Zn, Cu, Ca, P, K, Na, Mg, Mn	AAS	1-7<br>8-14<br>15-180	142	26<br>19<br>21	Full-term	Full expression	9-11 a.m	Pump	N.S.	N.S.	N.S.	18-40	N.S.	N.S.	N.S.	Not
[4]	Guiyang, Guizhou	Fe, Zn, Cu	AAS	5-7<br>42-56<br>90<br>180	120	N.S.	N.S.	N.S.	N.S.	N.S.	Han, Miao, Buyei	N.S.	Rural	22-32	N.S.	Primipara	N.S.	N.S.
[5]	Xiamen, Fujian	Zn, Cu, Mn	ICP-MS	3-5	200	200	Full-term	N.S.	N.S.	N.S.	N.S.	N.S.	N.S.	19-38	N.S.	N.S.	Monocyesis	N.S.
[6]	Beijing	Fe, Zn, Ca	AAS	42<br>90	100	50<br>50	Full-term	Fore-milk	9-11 a.m	Manual	N.S.	N.S.	N.S.	N.S.	N.S.	N.S.	N.S.	N.S.
[7]	Chaoyang, Liaoning	Fe, Zn, Cu	AAS	<12<br>13-30<br>31-60	1012		N.S.	N.S.	9-11 a.m	N.S.	N.S.	N.S.	N.S.	20-35	N.S.	Primipara	N.S.	N.S.

	Harbin, Heilongjiang<br>Liaocheng, Shandong<br>Wuzhong, Ningxia<br>Taicang, Jiangsu<br>Wuhan, Hubei<br>Nanning, Guangxi			61-90<br>91-120<br>121-150<br>151-180														
[8]	Nanjing, Jiangsu	Fe, Zn, Cu, Ca, Mg	ICP	3-6<br>40-50	80	80<br>40	Full-term	N.S.	N.S.	N.S.	N.S.	Yes	Urban	26.53	Vaginal (75%)	Primipara	N.S.	N.S.
[9]	Hohhot, Inner Mongolia	Fe, Zn, Ca, K, Na, Mg;<br>Se	AAS;<br>AFS	3-7<br>8-21<br>22-180	113	12<br>18<br>23	N.S.	N.S.	N.S.	N.S.	N.S.	N.S.	N.S.	N.S.	N.S.	N.S.	N.S.	N.S.
[10]	Baoding, Hebei	Zn, Cu, Mn	ICP-MS	3-5	206	206	Full-term	N.S.	N.S.	N.S.	N.S.	N.S.	Urban	20-35	Vaginal (62%)	N.S.	Monocyesis	N.S.
[11]	Shenzhen, Guangdong	Fe, Zn, Ca, K, Na, Mg	ICP-OES	30-60	235	235	N.S.	N.S.	N.S.	Manual	N.S.	N.S.	Urban (70.6%)	N.S.	N.S.	87%Primipara	Monocyesis	N.S.
[12]	Shanghai	Fe, Zn, Cu, Mn;<br>Na, K, Ca;<br>P;<br>Cl	AAS;<br>FES;<br>Plasma spectrometry<br>;Ion chromatography	8-10	120	120	Full-term	Fore-milk	9-11 a.m	Manual	N.S.	N.S.	Urban (75%)	22-36	N.S.	Primipara	N.S.	N.S.
[13]	Weihui, Henan	Fe, Zn, Cu;<br>Se	AAS;<br>AFS	7<br>21<br>90<br>210<br>300	27	27	Full-term	N.S.	N.S.	Manual, pump	N.S.	N.S.	Urban (51.8%)	N.S.	Vaginal	N.S.	N.S.	N.S.
[14]	Beijing	Fe, Zn, Ca;	AAS;<br>AFS	1	80	80<br>54	Full-term	N.S.	N.S.	N.S.	N.S.	N.S.	N.S.	N.S.	N.S.	Primipara	N.S.	N.S.

		Se																
[15]	Zhejiang	Fe, Zn, Cu, Ca, P, K., Na, Mg, Mn, Ba	ICP-AES	45<br>75<br>90<br>120<br>135<br>150<br>165<br>195<br>270<br>285	12	1<br>1<br>2<br>1<br>1<br>2<br>1<br>1<br>1<br>1	N.S.	N.S.	N.S.	N.S.	N.S.	N.S.	N.S.	N.S.	N.S.	N.S.	N.S.	N.S.
[16]	Shanghai	Fe, Zn, Cu, Ca, Mg	AAS	3<br>42	58	58	Full-term	N.S.	N.S.	N.S.	N.S.	N.S.	N.S.	N.S.	N.S.	N.S.	N.S.	N.S.
[17]	Guangdong	Fe, Zn, Cu, Ca	AAS	1<br>2<br>3<br>4<br>5<br>6<br>7	12	4<br>6<br>12<br>12<br>12<br>9<br>8	N.S.	N.S.	9-10 a.m	N.S.	N.S.	N.S.	N.S.	21-30	N.S.	Primipara	N.S.	N.S.
[18]	Beijing	Fe, Zn, Cu, Ca, Mg;<br>P	AAS;<br>Ammonium molybdate colorimetry	0-30<br>31-60<br>61-90<br>91-120<br>121-150<br>151-180<br>181-	132	9<br>20<br>24<br>24<br>22<br>23<br>10	Full-term	N.S.	10-12 a.m	N.S.	N.S.	N.S.	Urban (42.3%)	21-38	N.S.	Primipara	N.S.	Not
[19]	Nanning, Guangxi	Mn, Fe, Cu, Zn;<br>Ca	AAS;<br>FES	0-30<br>31-60<br>61-90<br>91-120<br>121-150<br>151-180	120	18<br>21<br>19<br>20<br>21<br>21	N.S.	Fore-milk	9-11 a.m	Manual	N.S.	N.S.	Urban	N.S.	N.S.	Primipara	N.S.	Not
[20]	Guangxi	Fe, Zn, Cu, Mn<br>Ca	AAS;<br>FES	0-30<br>31-60<br>61-90<br>91-120<br>121-15	240	35<br>42<br>40<br>41<br>41	N.S.	Fore-milk	9-11 a.m	Manual	Dong, Han	N.S.	N.S.	20-35	N.S.	N.S.	N.S.	Not

				0<br>151-18<br>0		42												
[21]	Lanzhou,<br>Gansu	Ca, Fe,<br>Zn	AAS	31-60<br>61-90<br>91-120<br>121-15<br>0<br>151-18<br>0	70	24<br>10<br>11<br>13<br>12	Full-ter<br>m	N.S.	N.S.	N.S.	N.S.	N.S.	N.S.	20-30	N.S.	Primipara	N.S.	N.S.
[22]	Tangshan<br>, Hebei	Se;<br>Ca;<br>P	AFS;<br>AAS;<br>Ammonium<br>molybdate<br>colorimetry	1-5<br>11	31	31<br>31	Full-ter<br>m	N.S.	N.S.	Manu<br>al	N.S.	N.S.	N.S.	24-30	N.S.	N.S.	N.S.	N.S.
[23]	Jinan,<br>Shandon<br>g	Fe, Zn,<br>Cu, Ca,<br>Mg	AAS	42	240	240	Full-ter<br>m	Fore-mil<br>k	9-10<br>a.m	Manu<br>al	N.S.	N.S.	N.S.	22-35	N.S.	Primipara	N.S.	N.S.
[24]	Guangxi	Fe, Zn,<br>Ca	AAS	15<br>30	240	50<br>50	Full-ter<br>m	Fore-mil<br>k	10 a.m	Manu<br>al	Zhuan<br>g	N.S.	N.S.	23-35	Vagina<br>l	N.S.	N.S.	Not
[25]	Tangshan<br>, Hebei	Fe, Zn,<br>Cu, Ca,<br>P, K,<br>Na,<br>Mg,<br>Mn	AAS,<br>ICP-MS	0-15<br>16-30<br>31-60<br>61-120<br>121-18<br>0<br>0-180	40	8<br>5<br>7<br>10<br>10<br>40	N.S.	N.S.	N.S.	N.S.	N.S.	N.S.	N.S.	N.S.	N.S.	N.S.	N.S.	N.S.
[26]	Beijing	Zn, Cu,<br>Ca,<br>Mg;<br>Se;<br>P	AAS;<br>AFS;<br>Ammonium<br>molybdate<br>colorimetry	3	65	65	N.S.	Full<br>expressi<br>on	N.S.	N.S.	N.S.	N.S.	N.S.	22-34	N.S.	N.S.	N.S.	N.S.
[27]	Nanning,<br>Guangxi	Fe, Zn,<br>Ca	AAS	30	50	50	Full-ter<br>m	Fore-mil<br>k	9-11<br>a.m	Manu<br>al	N.S.	N.S.	Urba<br>n	22-35	N.S.	N.S.	N.S.	Not
[28]	Nanning,<br>Guangxi	Zn, Cu,<br>Mg	AAS	30	50	50	Full-ter<br>m	N.S.	N.S.	N.S.	N.S.	N.S.	N.S.	23-35	N.S.	N.S.	N.S.	Not
[29]	Jinan,<br>Shandon<br>g	Fe, Zn,<br>Cu, Ca,<br>Mn	AAS	30-50	56	56	N.S.	N.S.	9:00 a.m<br>to 4:00<br>p.m	N.S.	N.S.	N.S.	N.S.	23-35	N.S.	N.S.	N.S.	N.S.
[30]	Hebei	Fe, Zn,<br>Cu, Ca,	ICP-MS	<30<br>30	150<br>90	168	N.S.	N.S.	N.S.	N.S.	N.S.	N.S.	Urba<br>n	20-36	Vagina<br>l (53%)	N.S.	N.S.	N.S.

		K, Na,<br>Mg		60<br>90<br>120	20<br>59<br>18								(76%,<br>)					
[31]	Lanzhou,<br>Gansu	Fe, Zn,<br>Cu, Ca,<br>K, Na,<br>Mg	N.S.	30-60	1215	5820	Full-ter<br>m	N.S.	N.S.	N.S.	Han	N.S.	N.S.	N.S.	N.S.	N.S.	Monocye<br>sis	N.S.
[32]	Enshi,<br>Hubei<br>Beijing<br>Liangsha<br>n,<br>Sichuan	Se	ICP-MS	15-20	60	60	N.S.	N.S.	N.S.	N.S.	N.S.	Yes	N.S.	N.S.	N.S.	N.S.	N.S.	N.S.
[33]	Laiwu,<br>Shandon<br>g	Fe, Zn,<br>Cu, Ca,<br>Mg	AAS	3-5<br>42	200	200<br>200	Full-ter<br>m	N.S.	N.S.	N.S.	N.S.	N.S.	N.S.	24-32	Vagina<br>l (50%)	Primipara<br>75%	N.S.	N.S.
[34]	Harbin,<br>Heilongji<br>ang<br>Beijing<br>Hohhot,<br>Inner<br>Mongolia<br>Chengdu,<br>Sichuan<br>Nanchan<br>g, Jiangxi<br>Shanghai<br>Guangzh<br>ou,<br>Guangdo<br>ng	Fe, Zn,<br>Cu, Ca,<br>Mn, K,<br>Na,<br>Mg;<br>P;<br>Se	AAS;<br>Spectrophotom<br>etry;<br>AFS	3-6<br>10-13<br>21-25	175	175<br>175<br>175	Full-ter<br>m	N.S.	9:00 a.m	N.S.	N.S.	N.S.	N.S.	20-40	N.S.	N.S.	N.S.	N.S.
[35]	Guangdo<br>ng	Zn, Cu	AAS	2-4	52	52	Full-ter<br>m	N.S.	N.S.	N.S.	N.S.	N.S.	N.S.	22-34	Vagina<br>l<br>(78.8%<br>)	Primipara<br>94%	N.S.	N.S.
[36]	Chaoyan<br>g,<br>Liaoning	Fe, Zn,<br>Cu, Ca,<br>Mg	AAS	0-30<br>31-60<br>61-90<br>91-120<br>121-15<br>0	164	19<br>28<br>27<br>29<br>26<br>27	N.S.	Full<br>expressi<br>on	9-11<br>a.m	Pump	N.S.	N.S.	N.S.	20-34	N.S.	N.S.	N.S.	N.S.

				151-18<br>0														
[37]	Lanzhou,<br>Gansu	Fe, Zn,<br>Cu, Ca,<br>Mg	ICP	3<br>90-180	N.S	27<br>29	N.S.	N.S.	N.S.	N.S.	N.S.	N.S.	N.S.	N.S.	N.S.	N.S.	N.S.	N.S.
[38]	Tianjin	Fe, Zn,<br>Ca, Cu	XRF	3-7	103	103	N.S.	N.S.	N.S.	Manu<br>al	N.S.	N.S.	N.S.	21-34	Vagina<br>l (53%)	Primipara<br>96%	N.S.	N.S.
[39]	Lanzhou,<br>Gansu	Fe, Zn,<br>Cu, Ca,<br>K, Na,<br>Mg	AAS	3<br>90	352	352<br>352	N.S.	N.S.	8-10<br>a.m	N.S.	N.S.	N.S.	N.S.	20-37	N.S.	N.S.	N.S.	N.S.
[40]	Nanjing,<br>Jiangsu	F	Electrode<br>potential<br>measurement	0-12<br>13-30<br>31-90<br>91-180<br>181-27<br>0	51	7<br>9<br>11<br>15<br>9	N.S.	N.S.	N.S.	N.S.	N.S.	N.S.	N.S.	N.S.	N.S.	N.S.	N.S.	N.S.
[41]	Wuhan,<br>Hubei	Cu, Fe,<br>Zn,<br>Mn, Ge	AAS	1-12<br>13-30<br>31-90	72	34<br>19<br>19	Full-ter<br>m	N.S.	8-10<br>a.m	N.S.	N.S.	N.S.	N.S.	22-32	Vagina<br>l	N.S.	N.S.	N.S.
[42]	Nanning,<br>Guangxi	Fe, Zn,<br>Cu, Ca,<br>Mg<br>Se, Mo,<br>Co, Si,<br>Ge	AAS	7<br>14<br>28<br>42<br>90<br>180	117	117<br>117<br>117<br>117<br>117<br>117	N.S.	N.S.	N.S.	N.S.	Zhuan<br>g,<br>Han,H<br>ui,<br>Manch<br>u,<br>Yao	N.S.	N.S.	23-35	N.S.	N.S.	N.S.	N.S.
[43]	Wuhan,<br>Hubei	Fe, Zn,<br>Cu,<br>Mn, Ge	AAS	1-12<br>13-30<br>60-180	176	34<br>19<br>19	N.S.	N.S.	N.S.	N.S.	N.S.	N.S.	N.S.	22-23	Vagina<br>l	Primipara	N.S.	N.S.
[44]	N.S.(Inne<br>r<br>Mongolia<br>?)	Fe, Zn,<br>Cu	AAS	1-19<br>11-29<br>21-69<br>61-120<br>121-18<br>0	N.S.	52<br>31<br>37<br>37<br>36	Full-ter<br>m	N.S.	N.S.	N.S.	N.S.	N.S.	N.S.	22-32	Vagina<br>l	N.S.	N.S.	N.S.
[45]	Weihui,<br>Henan	Fe, Zn,<br>Cu<br>Se	AAS<br>AFS	7<br>21<br>90	40	27<br>27<br>27	Full-ter<br>m	N.S.	8-12<br>a.m	Manu<br>al,<br>pump	N.S.	N.S.	N.S.	22-33	Vagina<br>l	N.S.	N.S.	N.S.

				210<br>300		27<br>27											
[46]	Weifang,<br>Shandon<br>g	Fe, Zn,<br>Cu, Ca,<br>Mg,<br>Mn, Ge	AAS	0-7<br>7-14<br>>14	N.S	68<br>60<br>64	N.S.	N.S.	N.S.	N.S.	N.S.	N.S.	N.S.	N.S.	N.S.	N.S.	N.S.
[47]	Jilin	Cu, Zn,<br>Mg	AAS	1-4<br>>5	N.S	39<br>12	N.S.	N.S.	N.S.	Manu<br>al	N.S.	N.S.	N.S.	21-38	N.S.	N.S.	N.S.
[48]	Tianjin	Fe, Zn,<br>Cu, Ca,<br>Mg	AAS	3-5<br>42	483	483<br>483	Full-ter<br>m	N.S.	N.S.	N.S.	N.S.	N.S.	N.S.	20-40	N.S.	N.S.	N.S.
[49]	Shanghai	Fe, Zn,<br>Cu, Ca,<br>Mg	AAS	3<br>42	67	63-67<br>31-33	Full-ter<br>m	N.S.	N.S.	N.S.	N.S.	N.S.	N.S.	23-35	N.S.	Primipara	Monocye<br>sis	N.S.
[50]	Baotou,<br>Inner<br>Mongolia	Fe, Zn,<br>Cu	AAS	1-10<br>11-20<br>21-60<br>61-120<br>121-18<br>0	193	52<br>31<br>37<br>37<br>36	Full-ter<br>m	N.S.	8-11<br>a.m	N.S.	N.S.	N.S.	N.S.	22-32	N.S.	Primipara	N.S.	N.S.
[51]	Tianjin	I	ICP-MS	7<br>28<br>84<br>168	N.S	301<br>142<br>85<br>60	N.S.	N.S.	N.S.	N.S.	N.S.	N.S.	N.S.	20-35	N.S.	N.S.	N.S.	N.S.
[52]	Enshi,<br>Hubei<br>Beijing<br>Liangsha<br>n,<br>Sichuan	Se	ICP-MS	90	264	264	N.S.	N.S.	N.S.	N.S.	N.S.	Yes	N.S.	N.S.	N.S.	N.S.	N.S.	N.S.
[53]	Hangzho<br>u,<br>Zhejiang	Fe	Iron kit	0-7<br>7-9<br>90-100<br>300-36<br>5	N.S	58<br>58<br>58<br>49	N.S.	N.S.	N.S.	N.S.	N.S.	N.S.	N.S.	N.S.	N.S.	N.S.	N.S.	N.S.
[54]	Hubei	Fe, Zn,<br>Cu, Ca,<br>Mg,<br>Mn	AAS	5	103	30	N.S.	N.S.	N.S.	N.S.	N.S.	Yes	N.S.	20-38	Vagina<br>l<br>(75.7%<br>)	Primipara<br>(90%)	N.S	N.S.

[55]	Wuhan, Hubei	Fe, Zn, Cu	AAS	3<br>7<br>14<br>21<br>28	9	9<br>9<br>8<br>7<br>6	Full-term	N.S.	8-10 a.m	Manual	N.S.	N.S.	N.S.	25.3±2.9	Vaginal	N.S.	N.S.	N.S.
[56]	Taiwan	Fe, Zn, Cu, Ca, Mg	ICP-AES	2-5<br>6-10<br>11-30<br>31-90<br>91-180<br>181-365	211	79<br>28<br>40<br>35<br>13<br>16	Full-term	N.S.	N.S.	N.S.	N.S.	N.S.	N.S.	20-30 (76.3%), >30 (23.7%)	N.S.	Primipara (39.8%)	Monocyesis	N.S.
[57]	Shanghai	Fe, Zn, Cu, Ca, Mg, P, K, Na, Cl, Mn	AAS	8-10	120	120	Full-term	N.S.	9-10 a.m	N.S.	N.S.	N.S.	Urban (75%)	22-36	N.S.	Primipara	Monocyesis	N.S.
[58]	Shanghai	Fe, Cu, Ca, Mg, P, K, Na, Mn;<br>Se;<br>Cl;<br>I	ICP;<br>GB5009.93-2010;<br>Potentiometric Titration;<br>HPLC	42	N.S	130	Full-term	N.S.	N.S.	Pump	N.S.	Yes	N.S.	N.S.	N.S.	N.S.	N.S.	Not
[59]	Yongjin, Gansu	I	Sandell-Kolthoff Methods	0-180<br>180-365	225	47<br>47	N.S.	N.S.	N.S.	N.S.	N.S.	Yes	N.S.	N.S.	N.S.	N.S.	N.S.	Not
[60]	Tianjin	I	ICP-MS	28<br>56<br>84<br>112<br>0-112	45	45<br>45<br>45<br>45<br>45	N.S.	N.S.	N.S.	N.S.	N.S.	Yes	N.S.	N.S.	N.S.	Primipara	N.S.	Not

Note: ICP-MS (Inductively Coupled Plasma Mass Spectrometry); AAS (Atomic Absorption Spectrometry); HPLC (High Performance Liquid Chromatography); AFS (Atomic Fluorescence Spectrometry); ICP-OES (Inductively Coupled Plasma-Optical Emission Spectrometer); ICP-AES (Inductively Coupled Plasma Atomic Emission Spectrometry); FES (Flame Atomic Emission Spectroscopy); XRF (X-ray Fluorescence Spectrometer.

Supplementary Table S3. List of all included studies for vitamins

References	City	Reported outcomes	Quantification Methods	Lactation (d)	Numbers of mothers	Numbers of samples	Full-term or pre-term	Fore-milk or hind-milk or full expression	Collection time	Manual or pump	Ethnic	Dietary supplements intake	Urban or rural	Maternal age (years of age)	Delivery	Primiparae or multiparae	Monocyesis or multiple gestation	Smoking or not
[61]	Beijing<br>Shanghai<br>Guangdong<br>Shandong<br>Zhejiang<br>Heilongjiang<br>Yunnan<br>Gansu<br>Inner Mongolia<br>Xinjiang<br>Guangxi	Vitamin B_1,<br>Vitamin B_2,<br>Vitamin B_6,<br>Nicotinic acid,<br>Pantothenic acid	UPLC-MS/MS	0-3<br>4-7<br>8-10<br>11-14<br>15-30<br>31-90<br>91-180<br>181-330	1778	211<br>275<br>194<br>222<br>224<br>164<br>223<br>212	Full-term	Full expression	9-11 a.m	N.S.	Han, Hui, Tibetan	Comparison of different diets	N.S.	20-35	N.S.	N.S.	Monocyesis	Not
[62]	Huangpu, Guangdong	Vitamin B1, B2, B6,<br>Nicotinic acid ,<br>Pantothenic acid,<br>Biotin	UPLC-MS/MS	4-7<br>8-10<br>11-15<br>16-30<br>31-60<br>31-100<br>101-300	N.S	30<br>28<br>23<br>27<br>20<br>24<br>31	Full-term	Full expression	9-11 a.m	N.S.	N.S	Comparison of different diets	N.S.	20-35	N.S.	N.S.	Monocyesis	Not
[63]	Beijing<br>Shanghai<br>Guangdong<br>Shandong<br>Zhejiang<br>Heilongjiang<br>Yunnan	Vitamin B1, B2, B6,<br>Nicotinic acid ,<br>Pantothenic acid,	UPLC-MS/MS	0-7<br>8-14<br>15-180<br>181-330	6419	486<br>416<br>611<br>232	Full-term	Full expression	9-11 a.m	N.S.	Han, Hui, Tibetan	Comparison of different diets	N.S.	20-35	N.S.	N.S.	Monocyesis	Not

	Gansu<br>Inner Mongolia<br>Xinjiang<br>Guangxi																	
[64]	Beijing<br>Suzhou, Jiangsu<br>Guangzhou, Guangdong	Vitamin B1, B2, B6<br>Nicotinic acid ,<br>Folic acid, Biotin	HPLC-MS	5-11<br>12-30<br>31-60<br>61-120<br>121-240	443	89<br>87<br>89<br>90<br>88	Full-term	Full expression	9-11 a.m	N.S.	N.S	N.S	N.S.	18-45	N.S.	N.S.	Monocyesis	Not
[65]	Beijing<br>Suzhou, Jiangsu<br>Guangzhou, Guangdong	Carotenoids (β-carotene , β-cryptoxanthin, lutein, lycopene, zeaxanthin)<br>Tocopherols(α-tocopherol, γ-tocopherol)	UHPLC	0-4<br>5-11<br>12-30<br>31-60<br>61-120<br>121-240	540	77<br>89<br>73<br>90<br>90<br>90	Full-term	Full expression	9-11 a.m	N.S.	N.S	N.S	N.S.	18-45	N.S.	N.S.	Monocyesis	Not
[66]	Chengdu, Sichuan<br>Shanghai<br>Tianjin<br>Guangzhou, Guangdong<br>Changchun, Jilin<br>Lanzhou, Gansu	Carotenoids (β-carotene , β-cryptoxanthin, lutein, lycopene, zeaxanthin)	HPLC	40-45	612	612	Full-term	Full expression	8-11 a.m	N.S.	N.S	N.S	N.S.	20-35	N.S.	N.S.	Monocyesis	N.S.
[18]	Beijing	Vitamin A,<br>Vitamin B_1,<br>Vitamin B_2;<br>Vitamin C;<br>Nicotinic acid	FS ;<br>2, 4-nitrobenzene trap method;<br>Microbiological method	0-30<br>31-60<br>61-90<br>91-120<br>121-150<br>151-180<br>181-	152	7<br>29<br>26<br>31<br>22<br>29<br>8	Full-term	N.S.	N.S.	N.S.	N.S.	N.S.	Urba (42.3 %)	21-38	N.S.	Primiparae	N.S.	Not

[19]	Nanning, Guangxi	Vitamin A	Microspectrofluorometry	0-30<br>31-60<br>61-90<br>91-120<br>121-150<br>151-180	120	18<br>21<br>19<br>20<br>21<br>21	N.S.	N.S.	N.S.	N.S.	N.S.	N.S.	Urban	N.S.	N.S.	Primiparae	N.S.	Not
[2]	Inner Mongolia	Vitamin C,<br>Vitamin B_1,<br>Vitamin B_2,<br>Vitamin B_6,<br>Nicotinic acid,<br>Folic acid,<br>Pantothenic acid,<br>Vitamin A,<br>Vitamin D,<br>Vitamin E,<br>Vitamin K	HPLC	3-7<br>8-21<br>22-180	66	7;<br>7<br>66	Full-term	Full expression	N.S.	N.S.	N.S.	N.S.	N.S.	28.3±3.9	N.S.	Primiparae (83.3%)	N.S.	N.S.
[67]	Hohhot, Inner Mongolia	Vitamin C,<br>Vitamin B_1,<br>Vitamin B_2,<br>Vitamin B_6,<br>Nicotinic acid,<br>Pantothenic acid,<br>Vitamin A,<br>Vitamin D,<br>Vitamin E,<br>Vitamin K	HPLC	3-7<br>8-21<br>22-180	53	5<br>20<br>38	N.S	N.S	N.S.	N.S.	N.S	N.S	N.S.	N.S.	N.S.	N.S.	N.S.	N.S.
[3]	Qiqihar, Heilongjiang	Vitamin A,<br>Vitamin E;<br>Vitamin B_1,<br>Vitamin B_2	HPLC;<br>FS	1-7<br>8-14<br>15-180	142	Vitamin A, Vitamin E, Vitamin B_1, Vitamin B_2 (n=4, 4, 26, 26); Vitamin B_1, Vitamin B_2 (n=23,	Full-term	Full expression	N.S.	N.S.	N.S.	N.S.	N.S.	18-40	N.S.	N.S.	N.S.	Not

						23);<br>Vitamin A,<br>Vitamin E,<br>Vitamin B_1,<br>Vitamin B_2<br>(n=4, 4, 23, 23)												
[68]	Hohhot, Inner Mongolia	Vitamin A,<br>Vitamin D,<br>Vitamin E	HPLC	3-7<br>16-30	70	Vitamin A,<br>Vitamin D,<br>Vitamin E<br>(n=37, 42, 36);<br>Vitamin A,<br>Vitamin D,<br>Vitamin E<br>(n=33, 43, 32)	Full-term	N.S	N.S.	N.S.	N.S	N.S	N.S.	N.S.	N.S.	N.S.	N.S.	N.S.
[69]	Nanning, Guangxi	Vitamin B_{12}	RIA	5	125	125	Full-term	N.S	N.S.	N.S.	Zhuang, Han, Yao	N.S	N.S.	25-30	N.S.	N.S.	N.S.	N.S.
[25]	Tangshan, Hebei	Vitamin C;<br>Vitamin B_1,<br>Vitamin B_2,<br>Nicotinic acid	FS;<br>HPLC	31-60<br>61-120<br>121-180<br>0-180	40	7<br>7<br>10<br>40	N.S.	N.S.	N.S.	N.S.	N.S.	N.S.	N.S.	N.S.	N.S.	N.S.	N.S.	N.S.
[70]	Hohhot, Inner Mongolia	Vitamin B_1,<br>Vitamin B_2,<br>Nicotinic acid	HPLC	1-7<br>16-30	84	Vitamin B_1,<br>Vitamin B_2,<br>Nicoti	Full-term	N.S	N.S.	N.S.	N.S	N.S	N.S.	N.S.	N.S.	N.S.	N.S.	N.S.

						nic<br>acid<br>(n=40,<br>17,<br>41);<br>Vitami<br>n B_1,<br>Vitami<br>n B_2,<br>Nicoti<br>nic<br>acid<br>(n=20,<br>33, 43)												
[71]	Zhousha<br>n,<br>Zhejiang	Vitamin A	HPLC	1-15<br>6-14<br>15-90<br>91-180	365	106<br>85<br>91<br>83	N.S.	N.S.	N.S.	N.S.	N.S.	Not	N.S.	N.S.	N.S.	N.S.	N.S.	N.S.
[20]	Guangxi	Vitamin A	Microspectrofluo<br>rometry	0-30<br>31-60<br>61-90<br>91-120-12<br>1-150<br>151-180	240	35<br>42<br>40<br>41<br>41<br>42	N.S.	N.S.	N.S.	N.S.	Dong,<br>Han	N.S.	N.S.	20-35	N.S.	N.S.	N.S.	Not
[72]	Shangha<br>i	α-tocopherol	HPLC	1-5<br>10-15<br>40-45	89	89	Full-te<br>rm	Full<br>express<br>ion	9-11<br>a.m	N.S.	N.S.	N.S.	N.S.	20-35	N.S.	N.S.	Monocy<br>esis	Not
[73]	Guilin,<br>Guangxi	Vitamin E	HPLC	2-4	12	12	N.S.	N.S.	8 a.m,<br>6 p.m	N.S.	N.S.	Not	N.S.	24-32	N.S.	N.S.	N.S.	Not
[74]	Hangzh<br>ou,<br>Zhejiang<br>Lanzhou<br>, Gansu<br>Beijing	Vitamin A,<br>α-tocopherol	HPLC	1<br>14<br>42	102	102	Full-te<br>rm	N.S.	10-11<br>a.m	N.S.	N.S.	Yes	N.S.	20-35	N.S.	N.S.	N.S.	Not
[75]	China	Vitamin A	HPLC	74±5		52	Full-te<br>rm	N.S.	N.S.	N.S.	N.S.	Not	N.S.	18-40	N.S.	N.S.	Monocy<br>esis	Not
[76]	Guilin,<br>Guangxi	α-tocopherol	HPLC	2-3<br>4-5<br>6-7<br>8-12	43	14<br>16<br>8<br>5	Full-te<br>rm	N.S.	N.S.	N.S.	N.S.	N.S.	N.S.	N.S.	N.S.	N.S.	N.S.	N.S.
[77]	Shangha	Tocopherol	HPLC	3±2	42	42	Full-te	Full	N.S.	N.S.	N.S.	N.S.	N.S.	18-40	N.S.	N.S.	Monocy	N.S.

	i	(α-tocopherol,<br>β-tocopherol)		13±2<br>43±2		42<br>42	rm	express<br>ion									esis	
[58]	Shangha<br>i	Vitamin B_1,<br>Vitamin B_2,<br>Vitamin B_6,<br>Nicotinic acid,<br>Vitamin A,<br>Vitamin K,<br>Vitamin E,<br>Vitamin D	HPLC	42	130	130	Full-te<br>rm	N.S.	N.S.	N.S.	N.S.	Yes	N.S.	N.S.	N.S.	N.S.	N.S.	Not
[78]	Wuxi,<br>Jiangsu	Vitamin E (α-、β-、<br>γ-、δ-tocopherol)	HPLC	1-7<br>8-14<br>>15	103	103<br>103<br>103	Full-te<br>rm	N.S.	N.S.	N.S.	N.S.	Yes	N.S.	27.9±3<br>.2	N.S.	N.S.	Monocy<br>esis	N.S.

Note: UPLC-MS/MS (Ultra Performance Liquid Chromatography-Tandem Mass Spectrometry); HPLC-MS (High Performance Liquid Chromatography-Mass Spectrometry); UHPLC (Ultra-High Performance Liquid Chromatography); HPLC (High Performance Liquid Chromatography); FS (Fluorescence spectrophotometer); RIA (Radioimmunoassay).

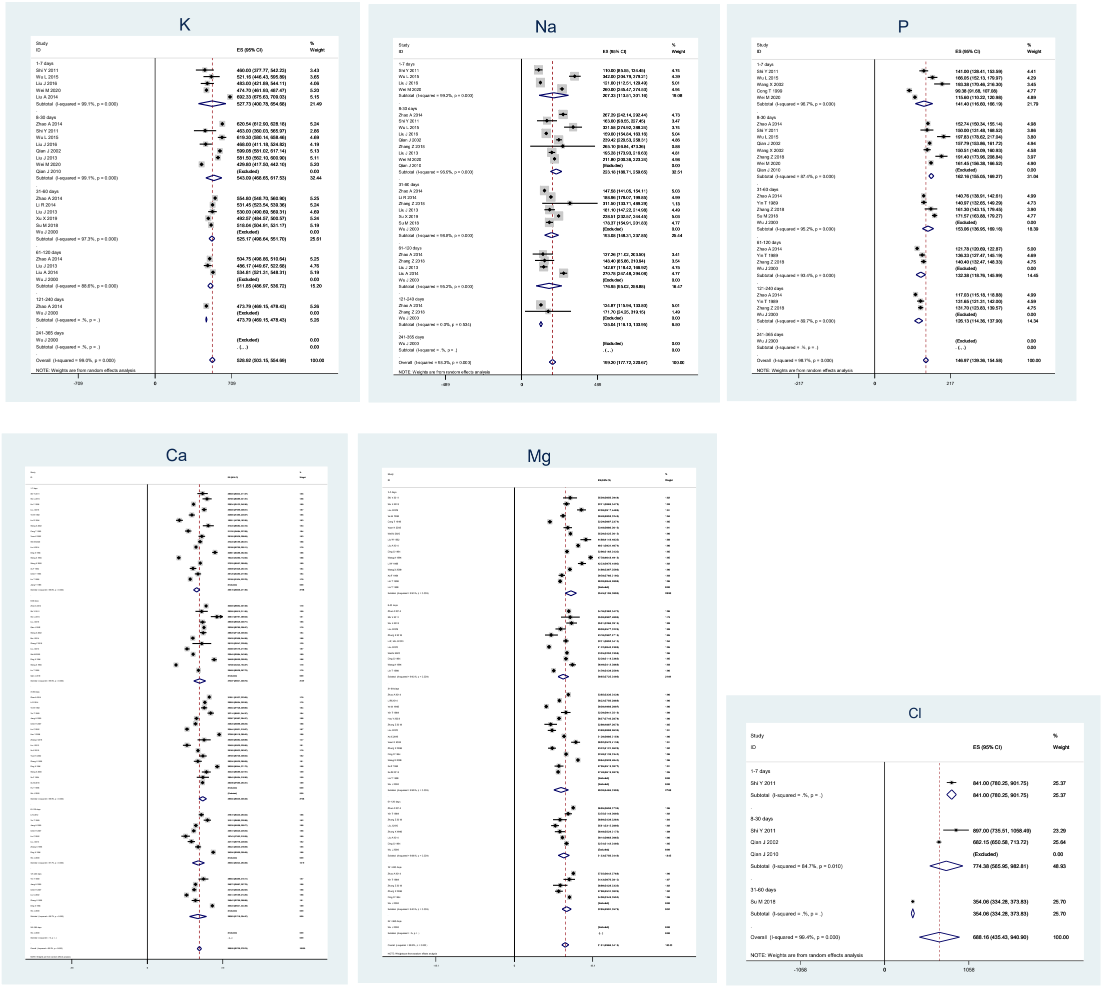

Supplementary Figure S1. Forest plot of comparison of macro mineral elements concentrations

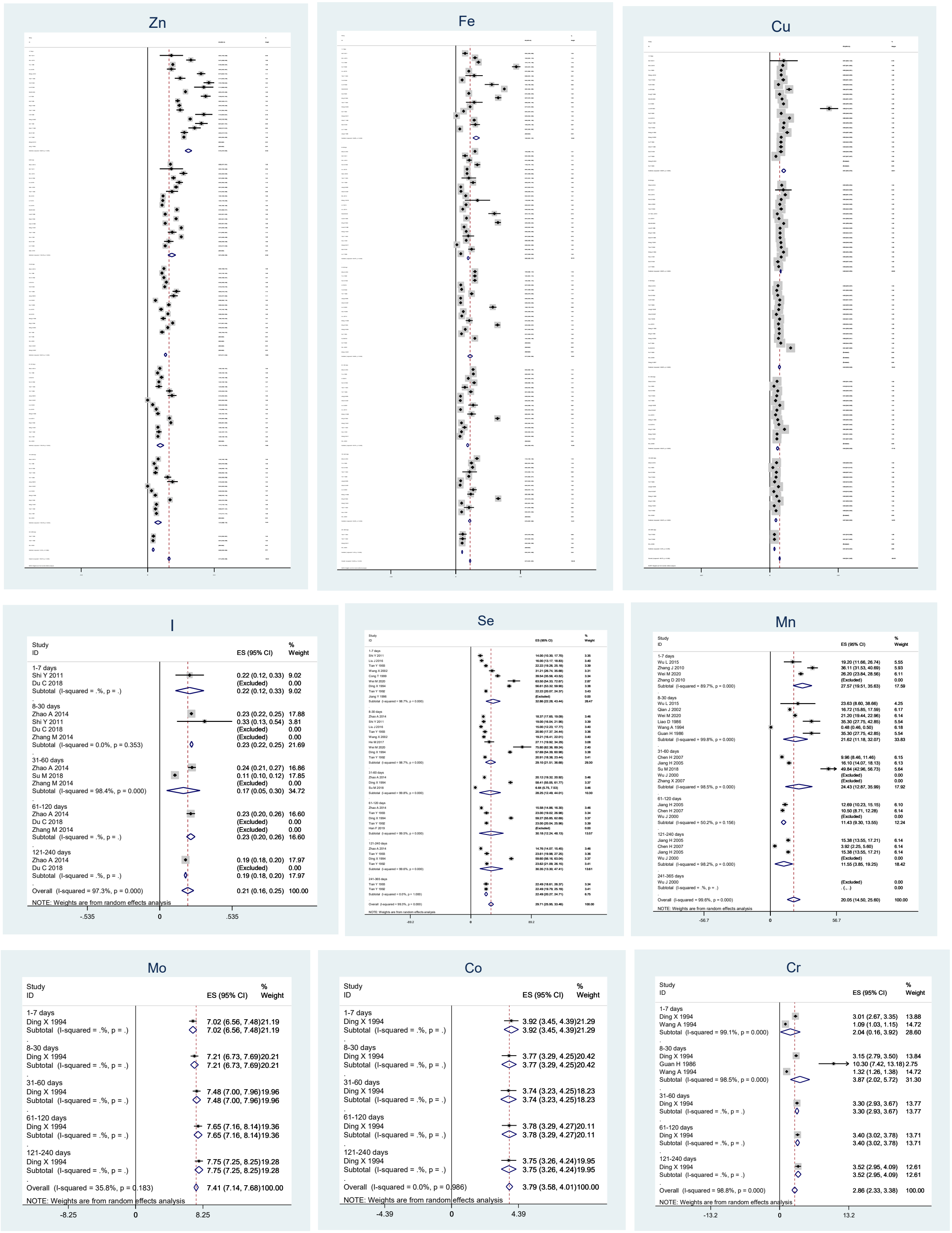

Supplementary Figure S2. Forest plot of comparison of trace elements concentrations

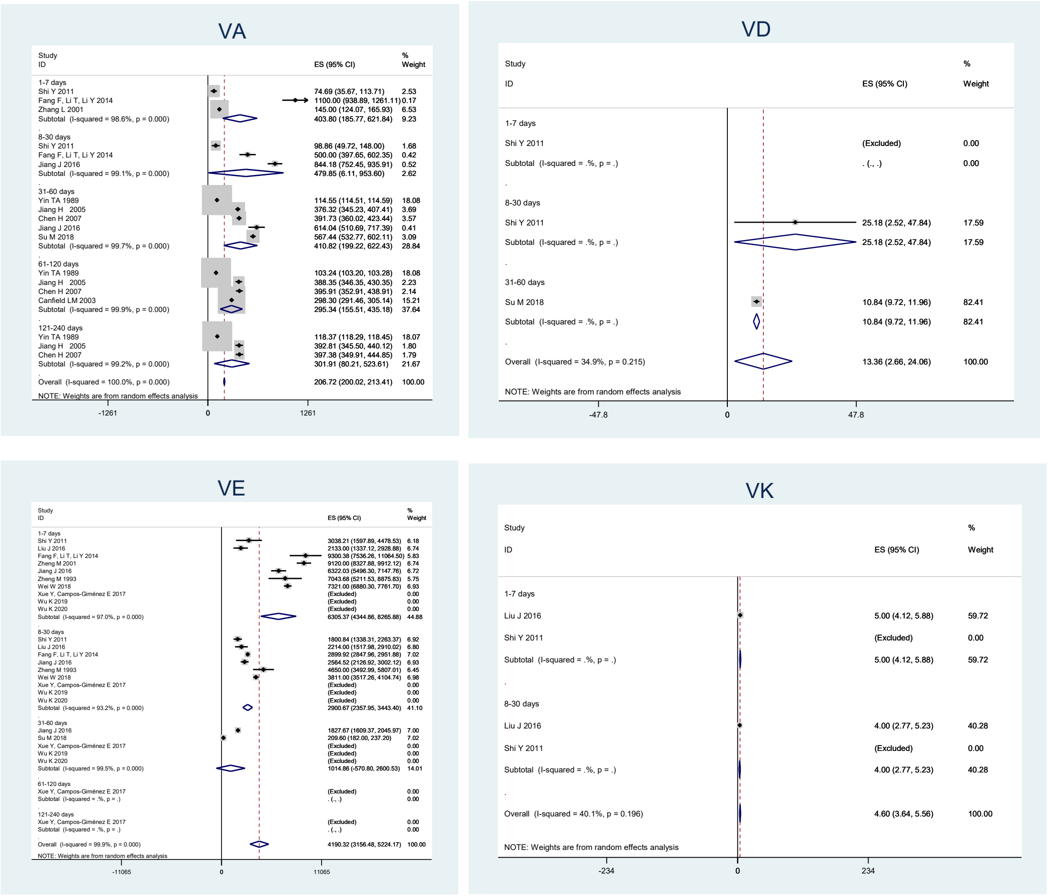

Supplementary Figure S3. Forest plot of comparison of fat-soluble vitamins concentrations

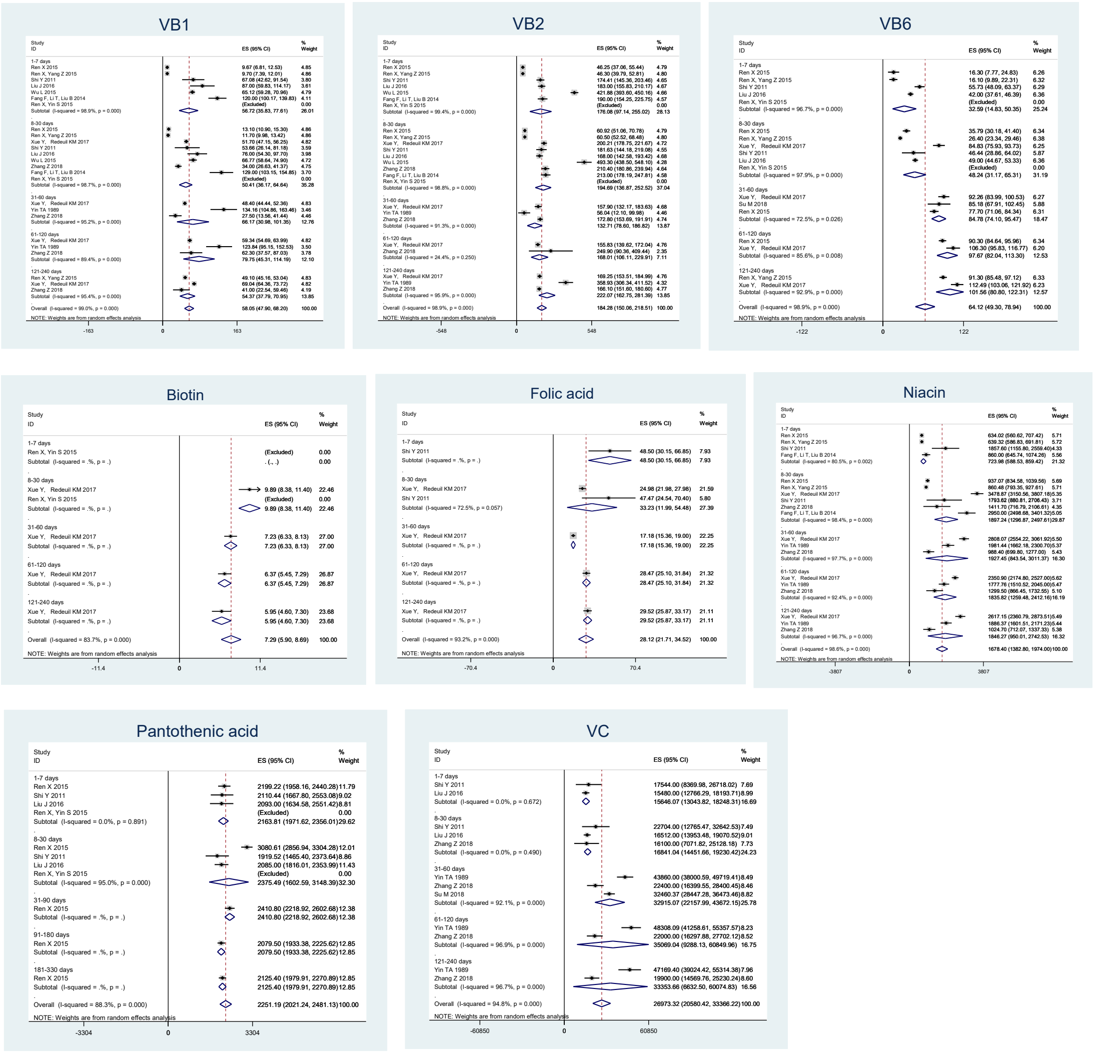

Supplementary Figure S4. Forest plot of comparison of water-soluble vitamins concentrations

Supplementary Table S4. Longitudinal changes in mineral and vitamin concentrations in human milk in Chinese population

	1-7 days			8-30 days			31-60 days			61-120 days			120-240 days			240-365 days		
	Mean	SE	n	Mean	SE	n	Mean	SE	n	Mean	SE	n	Mean	SE	n	Mean	SE	n
K	610.7	26.6	572	546.5	19.2	1022	512.5	12	2044	515.2	18.4	873	473.8	2.4	444	NA	NA	NA
Na	257.3	18.8	220	238.1	10.3	1053	209	5.2	2051	191.1	19	883	125.9	7.6	454	NA	NA	NA
Ca	260.2	6.7	1873	273.5	8.7	1568	292.2	5.7	3315	274.3	15.7	489	284.2	19.8	312	NA	NA	NA
P	125	7.7	304	157.5	6	976	147.7	6.9	601	123.5	5.9	502	118.8	5.7	499	NA	NA	NA
Mg	35.3	0.9	1795	32.4	1.1	1317	32.4	0.6	3228	32.9	1.1	1104	35.5	1.5	679	NA	NA	NA
Cl	841	31	7	694	64	127	354.1	32.2	130	NA	NA	NA	NA	NA	NA	NA	NA	NA
Zn	5.38	0.15	1685	3.13	0.1	1719	2.44	0.05	3215	1.78	0.05	1833	1.41	0.05	1293	0.7	0.14	54
Fe	0.996	0.0419	1345	1.0012	0.0421	1592	0.7674	0.0242	2130	0.862	0.0339	1451	1.0199	0.0417	1256	0.3567	0.0522	103
Cu	0.6045	0.0175	2052	0.5079	0.0157	1663	0.3999	0.0107	1853	0.3542	0.0109	1710	0.2897	0.0107	1226	0.2096	0.0377	54
I	0.221	0.054	7	0.2355	0.0127	451	0.2095	0.0147	574	0.2291	0.0162	444	0.1902	0.0047	444	NA	NA	NA
Se	0.0426	0.0027	311	0.0325	0.0018	898	0.0241	0.0012	691	0.0245	0.0013	615	0.0241	0.0013	615	0.0225	0.0039	54
Mn	0.0308	0.002	407	0.0192	0.0012	587	0.0368	0.0046	183	0.0108	0.0015	120	0.0132	0.002	104	NA	NA	NA
Mo	0.007	0.0002	117	0.0072	0.0005	234	0.0075	0.0002	117	0.0077	0.0003	117	0.0078	0.0003	117	NA	NA	NA
Co	0.0039	0.0002	117	0.0038	0.0003	234	0.0037	0.0003	117	0.0038	0.0002	117	0.0038	0.0003	117	NA	NA	NA
Cr	0.0023	0.0002	185	0.0032	0.0003	313	0.0033	0.0002	117	0.0034	0.0002	117	0.0035	0.0003	117	NA	NA	NA
Sr	0.8669	NA	117	0.8359	NA	234	0.8153	NA	117	0.7946	NA	117	0.743	NA	117	NA	NA	NA
F	NA	NA	NA	0.0804	NA	9	NA	NA	NA	0.0636	NA	15	NA	NA	NA	NA	NA	NA
Ba	NA	NA	NA	NA	NA	NA	0.01	NA	1	0.0103	NA	4	0.0012	NA	5	0.03	NA	2
VA	867.3	74.1	261	727.5	72.9	142	505.2	40.2	325	305.8	30	222	310	36	181	NA	NA	NA
VD	NA	NA	NA	25.2	11.6	7	10.8	1	140	NA	NA	NA	NA	NA	NA	NA	NA	NA
VE	7071.8	483.8	303	3107.8	225.1	259	2609.5	258.4	232	NA	NA	NA	NA	NA	NA	NA	NA	NA
VK	5	0.4	5	4	0.6	10	NA	NA	NA	NA	NA	NA	NA	NA	NA	NA	NA	NA

VB1	16	1.3	1050	19.2	1	1208	67.1	7.8	125	74.8	8	131	54.2	3.4	330	NA	NA	NA
VB2	59.7	3.7	1027	81.8	4	1211	135.1	16.6	125	165.2	20	100	213.5	27	128	NA	NA	NA
VB6	16.6	2.3	984	36.3	1.8	1160	83.6	5.7	383	94.9	6	313	97.1	6	320	NA	NA	NA
Biotin	NA	NA	NA	9.9	0.8	87	7.2	0.5	89	6.4	0.5	90	6	0.7	88	NA	NA	NA
Folic acid	48.5	9.4	7	26.7	3.3	94	17.2	0.9	89	28.5	1.7	90	29.5	1.9	88	NA	NA	NA
Niacin	654	31.1	1020	1174.3	51.5	1198	2514.4	250.9	125	2075.8	199.3	157	2241.1	220.9	157	NA	NA	NA
Pantothenic acid	2196.9	165.1	498	3053.1	180.6	657	2410.8 (31-90 days)	97.9	164	2079.5 (91-180 days)	74.6	223	2125.4 (181-330 days)	74.2	212	NA	NA	NA
VC	16684	5654.4	12	18388.5	4450.2	22	34027.6	3705.1	166	44495.3	8047.1	69	43217.3	7831.2	69	NA	NA	NA

Supplementary Table S5. Shapiro-Wilk test

Mineral	*W*	*p*	Vitamin	*W*	*p*
K	0.936	0.12	VA	0.741	2.24×10^{-6}
Na	0.931	0.06	VD	0.782	0.07
Ca	0.981	0.22	VE	0.885	0.03
P	0.971	0.64	VB1	0.907	0.02
Mg	0.979	0.33	VB2	0.825	4.87×10^{-4}
Cl	0.896	0.39	VB6	0.923	0.11
Zn	0.919	1.70×10^{-6}	Biotin	0.866	0.28
Fe	0.842	8.67×10^{-11}	Folic acid	0.885	0.29
Cu	0.638	1.68×10^{-15}	Niacin	0.931	0.09
I	0.939	0.60	Pantothenic acid	0.918	0.27
Se	0.820	1.81×10^{-5}	VC	0.881	0.04
Mn	0.901	0.02			
Mo	0.944	0.69			
Co	0.772	0.03			
Cr	0.678	7.81×10^{-4}			

Note: $p>0.05$ indicates that the normal distribution is satisfied

Supplementary Table S6. Comparison of recommended dietary intakes

	This review-Chinese human milk						DRIs for China		DRIs for America	
	1-7 days	8-30 days	31-60 days	61-120 days	1-120 days	121-240 days	0-6 month	7-12 month	0-6 month	7-12 month
K (mg/d)	458	410	384	386	400	355	400	600	400	860
Na (mg/d)	193	179	157	143	161	94	80	180	110	370
Ca (mg/d)	195	205	219	206	209	213	200	350	200	260
P (mg/d)	94	118	111	93	108	89	105	180	100	275
Mg (mg/d)	26	24	24	25	25	27	20	65	30	75
K/Na	2.4	2.3	2.4	2.7	2.5	3.8	-	-	-	-
Ca/P	2.1	1.7	2.0	2.2	1.9	2.4	-	-	-	-
Ca/Mg	7.5	8.5	9.1	8.2	8.5	7.9	-	-	-	-
K/Mg	17.6	17.1	16.0	15.4	16.2	13.1	-	-	-	-
P/Mg	3.6	4.9	4.6	3.7	4.4	3.3	-	-	-	-
Cl (mg/d)	631	521	266	-	398	-	120	450	180	570
Zn (mg/d)	4.04	2.35	1.83	1.34	2.27	1.06	1.5	3.2	2	3
Fe (mg/d)	0.75	0.75	0.58	0.65	0.67	0.76	0.3	10	0.27	11
Cu (mg/d)	0.45	0.38	0.30	0.27	0.35	0.22	0.3	0.3	0.20	0.22
I (μg/d)	166	177	157	172	168	143	85	115	110	130
Se (μg/d)	32	24	18	18	22	18	15	20	15	20
Mn (mg/d)	0.023	0.014	0.028	0.008	0.018	0.010	0.01	0.7	0.003	0.6
Mo (μg/d)	5.25	5.40	5.63	5.78	5.49	5.85	3	6	2	3
Cr (μg/d)	1.73	2.40	2.48	2.55	2.27	2.63	0.2	5	0.2	5.5
F (mg/d)	-	0.06	-	0.05	0.05	-	0.01	0.23	0.01	0.5

VA (μg RAE/d)	650	546	379	229	443	233	300	350	400	500
VD (μg/d)	-	19	8	-	9	-	10	10	10	10
VE (mg α-TE/d)	5.30	2.33	1.96	1.85	3	1.91	3	4	4	5
VE/VA	8.2	4.3	5.2	8.1	7.3	8.2	-	-	-	-
VK (μg/d)	3.8	3.0	-	-	3. 3	-	2.0	10	2.0	2.5
VB_1 (mg/d)	-	-	0.1	0.1	0. 1	-	0.1	0.3	0.2	0.3
VB_2 (mg/d)	-	0.1	0.1	0.1	0. 1	0.2	0.4	0.6	0.3	0.4
VB_6 (mg/d)	-	-	0.1	0.1	0. 1	0.1	0.1	0.3	0.1	0.3
Pantothenic acid (mg/d)	1.6	2.3	1.8	1.6	1.9	1.6	1.7	1.9	1.7	1.8
Niacin (mg/d)	0.5	0.9	1.9	1.6	0.8	1.7	1.0	2.0	2	4
Folic acid (μg DFE/d)	36	20	13	21	18	22	65	100	65	80
Biotin (μg/d)	-	7	5	5	6	5	5	10	5	6
VC (mg/d)	13	14	26	33	26	32	40	40	40	50

References

1. Zhao, A.; Ning, Y.; Zhang, Y.; Yang, X.; W, J.; Li, W.; Wang, P. Mineral Compositions in Breast Milk of Healthy Chinese Lactating Women in Urban Areas and Its Associated Factors. *Chinese Medical Journal* **2011**, *127*: 2643-2648.
2. Shi, Y.; Sun, G.; Zhang, Z.; Deng, X.; Kang, X.; Liu, Z.; Ma, Y.; Sheng, Q. The Chemical Composition of Human Milk from Inner Mongolia of China. *Food Chemistry* **2011**, *127*, 1193–1198, doi:10.1016/j.foodchem.2011.01.123.
3. Wu, L. Human milk composition study and the comparative study on key components in breast milk and infant formula[D], Chinese Center for Disease Control and Prevention, 2015. (in Chinese)
4. Yu, L.; Ma, M. Determination and Analysis of Trace Element Content in Plasma and Breast Milk of Han, Miao, and Buyi Pregnant Women in the Suburbs of Guiyang City. *Guizhou Medical Journal* **1998**, 230–232. (in Chinese)
5. Zhang, D.; Chen, G.; Xu, J.; Zou, X.; Yan, C. Correlation of trace elements between maternal milk and blood,and inter-element correlations in human milk. *Chinese Journal of Child Health Care* **2010**, *18*, 199–201. (in Chinese)
6. Li, N.; He, Q.; Ren, C.; Lin, F.; Li, H.; Zhang, W. Study on change characters and correlations of iron, zinc and calcium in milk and blood of mother and infant at different stages. *Journal of Hygiene Research* **2012**, *41*, 225–227, doi:10.19813/j.cnki.weishengyanjiu.2012.02.014. (in Chinese)
7. Sun, G.; Chen, X.; Yang, Y. A study in zinc, copper and iron contents in human milk. *Acta Nutrimenta Sinica* **1994**, 61–66. (in Chinese)
8. Hu, Y.; Luo, Y.; Dai, L.; Zhou, Z.; Cao, M.; Chen, Y.; Wang, J. Analysis of iron, zinc, copper, calcium, magnesium content in breast milk during different lactation periods. *Chinese Journal of Practical Gynecology and Obstetrics* **1998**, 47–48. (in Chinese)
9. Liu, J. Study on the Mineral Contents of 113 Human Milks in Huhhot. *Food Research and Development* **2016**, *37*, 117–119. (in Chinese)
10. Zhang, J.; Li, Y.; Liu, X.; Liu, W.; Wang, C. Levels of mineral elements and heavy metal pollution in human breast milk in Baoding city. *Journal of Hebei Medical University* **2010**, *31*, 1326–1328. (in Chinese)
11. Li, R.; Qi, C.; Jiang, J.; Zhang, H.; Li, S.; Lin, K.; Jiang, Y.; Zhou, J.; Lin, X.; Zhang, J. Evaluation of nutrient level and its factors of breast milk in Shenzhen City. *Journal of Hygiene Research* **2014**, *43*, 550-555+561, doi:10.19813/j.cnki.weishengyanjiu.2014.04.005. (in Chinese)
12. Qian, J.; Wu, S.; Zhang, W.; Cao, L.; Yang, H.; Ao, L. An investigation of nutrients of human milk in Shanghai area. *Shanghai Medical Journal* **2002**, 396–398. (in Chinese)
13. Tian, Y.; Yan, L.; Gao, J.; Tian, H.; Pu, X.; Li, S.; Li, J. The difference of the trace elerent contents of lactation breast milk and growth development of their breast-fed infants between city and rural area. *Journal of Xinxiang Medical University* **1993**, 284–287. (in Chinese)
14. Jiang, Y.; Lv, Z.; Fan, H.; Qian, D.; Si, W.; Zhang, Y. Effects of Two Different Rooming-in On Components of Breast Milk--A studies about Zinc. Iron ,Calcium and Selenium in colostrum. *Studies of Trace Elements and Health* **1996**, 20–21. (in Chinese)

15. Wu, J.; Su, J.; Zhu, Y. Comparison of trace elements in some human milk and milk samples. *Environmental Pollution & Control* **2000**, 40–41, doi:10.15985/j.cnki.1001-3865.2000.01.018. (in Chinese)
16. Ye, W.; Zhang, C. Investigation of trace element content in breast milk. *Laboratory Medicine* **1992**, 249. (in Chinese)
17. Liu, R.; Wang, M.; Shi, L.; Zheng, D. Paired analysis of trace elements in maternal hair, offspring hair, and breast milk. *Journal of Guangdong Medical University* **1994**, 119–120. (in Chinese)
18. Yin, T.; Liu, D.; Li, L.; Wang, W.; Yan, H.; Ji, Y.; Xu, Q.; Fu, A.; Bai, J.; Dai, J.; et al. Studies of the relationship between the nutritional status of lactating mothers and milk composition as well as the milk intake and growth of their infants in Beijing V· Essential inorganic elementsand vitamins in human milk. *Acta Nutrimenta Sinica* **1989**, 233–239. (in Chinese)
19. Jiang, H.; Chen, H.; Wang, Y.; Wang, X.; Huang, Y.; Yao, Q.; Meng, D. Analysis of Nutrient Content in Breast Milk of Nurses in Nanning City. *Journal of Guangxi Medical University* **2005**, 42–44, doi:10.16190/j.cnki.45-1211/r.2005.05.015. (in Chinese)
20. Chen, H.; Jiang, H.; Yang, W.; Wang, X.; Huang, Y. Investigation and Analysis of Dietary and Nutrient Content in Milk of Dong and Han Breast Mothers in Sanjiang County, Guangxi and Nanning City. *Journal of Guangxi Medical University* **2007**, 644–647, doi:10.16190/j.cnki.45-1211/r.2007.04.044. (in Chinese)
21. Liu, C.; Bai, L.; Liang, B.; Chen, Y. Relations between Height and Weight of Infants and the Content of Ca，Fe，Zn in Maternal Milk. *Guangdong Trace Elements Science* **2002**, 36–38, doi:10.16755/j.cnki.issn.1006-446x.2002.04.003. (in Chinese)
22. Wang, X.; Wang, C. Comparison of calcium, phosphorus, selenium content and GPX activity in normal breast milk. *Chinese Journal of Public Health* **2002**, 99. (in Chinese)
23. Hou, Y.; Yu, S.; Zheng, X.; Fu, J. Analysis of the composition of milk from 240 lactating mothers in Jinan City. *Maternal and Child Health Care of China* **2008**, 241–243. (in Chinese)
24. Mo, J.; Huang, Y.; Zhou, R.; Wei, L.; Yuan, M. Influence of Individual Diet Instruction on Thelastria Diettary Pattern and Calcium Iron Zinc Contents in Breast Milk in Zhuang Population. *Guangxi Medical Journal* **2014**, *36*, 776–779. (in Chinese)
25. Zhang, Z.; Sun, Y.; Tian, Y.; Zhang, Z.; He, Q. Analysis of Nutritional Ingredients in Breast Milk on Tangshan County. *Food Research and Development* **2018**, *39*, 175–179. (in Chinese)
26. Cong, T.; Zhao, L.; Yuan, Y.; Han, W.; Li, Z.; Zhang, Y. The analysis of mineral and trace elements in 65 cases breast colostrum and its clinical nutritive significance. *Chinese Journal of Clinical Nutrition* **1999**, 12–14. (in Chinese)
27. Li, F. The Research on the Influence of Dietnursing on the Concentrations of Calcium Iron and Zinc in Breast Milk of Lactating Women[D], Guangxi Medical University, 2013. (in Chinese)
28. Li, F.; Mo, J. Influence of dietary intervention on the dietary nutrition status of lactating women and the concentrations of zinc，copper，and magnesium in breast milk. *Chinese Journal of New Clinical Medicine* **2013**, *6*, 583–586. (in Chinese)

29. Zhang, X.; Yu, H.; Leng, J.; Wang, S.; Wang, Y. A Study on the Dietary and Mineral Content of Breast Milk. *Journal of Hygiene Research* **2007**, 393–394. (in Chinese)
30. Liu, J. A survey and study on the dynamic changes of nutritional components in breast milk and the influence factors of infant growth and development in Shijiazhuang[D], Hebei Medical University, 2013. (in Chinese)
31. Xu, X.; Yang, L.; Yang, D.; Wu, Q.; Wang, W.; Zhou, M. Energy Supply Nutrients and Mineral Composition Analysis in Breast Milk in Lanzhou Urban. *Chinese Journal of Clinical Nutrition* **2019**, *27*, 62–64.
32. He, M. Study on the Content and Morphology of Selenium in Human Milk[D], Chinese Journal of Neonatology, 2017. (in Chinese)
33. Yuan, K.; Yuan, H.; Fu, M.; Chen, L. Detection and analysis of trace element content in maternal milk and serum. *Occupation and Health* **2002**, 123–124. (in Chinese)
34. Wei, M.; Deng, Z.; Liu, B.; Ye, W.; Fan, Y.; Liu, R.; Li, J. Investigation of Amino Acids and Minerals in Chinese Breast Milk. *J Sci Food Agric* **2020**, *100*, 3920–3931, doi:10.1002/jsfa.10434.
35. Li, X.; Niu, G.; Yu, Y.; Shen, H.; Wang, J. Determination of zinc and copper content in maternal serum and colostrum. *Journal of New Medicine* **1998**, 84–85. (in Chinese)
36. Zhang, X.; Wang, X.; Wang, A.; Sun, G.; Yang, Y.; Chen, X. Determination of Five Elements in Human Milk in Chaoyang City. *Medical Journal of Liaoning* **1996**, 55–56. (in Chinese)
37. Liu, W.; Bai, C.; Ni, J. Analysis and nutritional evaluation of five trace elements in colostrum, mature milk, and milk. *Studies of Trace Elements and Health* **1992**, 32–33. (in Chinese)
38. He, Z.; Sun, D.; Yang, H.; Zhao, X. Analysis of zinc, copper, iron, and calcium content in healthy lactating mothers' colostrum (report of 103 cases). *Progress in Obstetrics and Gynecology* **1995**, 36–37. (in Chinese)
39. Liu, A.; Zhang, G.; Zhao, C. Comparative analysis of several elements in colostrum and three months postpartum milk of lactating women in Lanzhou City. *Gansu Science and Technology* **2014**, *30*, 124–125. (in Chinese)
40. Xie, H.; Li, J.; Zhu, Z.; Si, C.; Wu, C. Study on the Changes of Fluorine in Human Milk with Breast-feeding Time. *Journal of Nanjing Military Medical College* **1994**, 102–103. (in Chinese)
41. Liao, D.; Guan, H.; Wang, G.; Cai, R.; Li, J. Comparison of the contents of Copper, Iron, Zinc, Manganese and Chromium between Human Milk and Cow's Milk. *Acta Nutrimenta Sinica* **1986**, 360–365. (in Chinese)
42. Ding, X.; He, Y.; Lin, W.; Lin, K.; Luo, J.; Huang, W.; Huang, Y.; Chen, Z.; Tang, Q.; Shi, W. Determination, Analysis and Clinical Application of 10 Elements in Breast Milk. *Studies of Trace Elements and Health* **1994**, 49–51. (in Chinese)
43. Guan, H.; Dai, Q.; Wu, J.; Liao, D. Study on Anti infective Factors and Trace Elements in Breast Milk. *Chinese Journal of Neonatology* **1986**, 250–252. (in Chinese)
44. Wang, Y.; Li, W.; Wang, W.; Bai, S. The Content Change of Cu，Fe and Zn in Breast Milk and Comparison with the Cow Milk. *Journal of Inner Mongolia Minzu University(Natural Sciences)* **2001**, 249–251, doi:10.14045/j.cnki.15-1220.2001.03.008. (in

Chinese)
45. Tian, Y.; Yang, L.; Gao, J.; Li, S.; Ma, L.; Tian, H.; Li, J. Dynamic Study of Trace Elements Contents in Human Milk and the Relationship between Trace Elements and Infants Growth. *Journal of Xinxiang Medical University* **1992**, 291-294+354. (in Chinese)
46. Wang, A.; Ding, S.; Xu, X.; Li, Z.; Yang, Y. Study on Trace Elements in Human Milk. *Studies of Trace Elements and Health* **1994**, 38-39+35. (in Chinese)
47. Li, W.; Mo, X. The Content of Cu, Zn, and Mg in Human Milk. Journal of Jilin. *Journal of Jilin University (Medicine Edition)* **1988**, 56–58, doi:10.13481/j.1671-587x.1988.01.023. (in Chinese)
48. Wang, X.; Liu, Y.; Li, L.; Zhao, Z. Analysis of Element Content in Milk of 483 Maternal Women in Tianjin. *Chinese Journal of Practical Gynecology and Obstetrics* **2000**, 55–56. (in Chinese)
49. Xu, F.; Ye, W. The content of trace elements in colostrum and mature milk of normal full-term postpartum women. *Academic Journal of Naval Medical University.* **1994**, 194–196, doi:10.16781/j.0258-879x.1994.02.034. (in Chinese)
50. Huo, J.; Yang, C.; Liu, S. Changes in copper, iron, and zinc content in breast milk during different lactation stages and their relationship with trace element content in maternal plasma. *Journal of Hygiene Research* **1991**, *20*, 41–43, doi:10.19813/j.cnki.weishengyanjiu.1991.02.015. (in Chinese)
51. Du, C.; Wang, C.; Zhang, Y.; Fan, L.; Wang, W.; Chen, W.; Shen, J.; Zhang, W. The iodine status of lactating women and the influence on infants in Tianjin City. *Journal of Hygiene Research* **2018**, *47*, 543–547, doi:10.19813/j.cnki.weishengyanjiu.2018.04.008. (in Chinese)
52. Han, F.; Liu, L.; Lu, J.; Chai, Y.; Zhang, J.; Wang, S.; Sun, L.; Wang, Q.; Liu, Y.; He, M.; et al. Calculation of an Adequate Intake (AI) Value and Safe Range of Selenium (Se) for Chinese Infants 0–3 Months Old Based on Se Concentration in the Milk of Lactating Chinese Women with Optimal Se Intake. *Biol Trace Elem Res* **2019**, *188*, 363–372, doi:10.1007/s12011-018-1440-9.
53. Wang, H.-J.; Hua, C.-Z.; Ruan, L.-L.; Hong, L.-Q.; Sheng, S.-Q.; Shang, S.-Q. Sialic Acid and Iron Content in Breastmilk of Chinese Lactating Women. *Indian Pediatr* **2017**, *54*, 1029–1031, doi:10.1007/s13312-017-1206-z.
54. Chen, T.; Li, W.; Zhang, C.; Gao, Z. Clinical and Experimental Study of Yangxueshengru Oral Liquor in Promoting Puerperal Breast Milk Secretion. *Chinese Journal of Integrated Traditional and Western Medicine* **1995**, 528–531. (in Chinese)
55. Dai, D.; Tang, Z. Copper, Iron and Zinc Content of Preterm and Term Human Milk and Cow's Milk. *Studies of Trace Elements and Health* **1992**, 25–28. (in Chinese)
56. Lin, T.-H.; Jong, Y.-J.; Chiang, C.-H.; Yang, M.-H. Longitudinal Changes in Ca, Mg, Fe, Cu, and Zn in Breast Milk of Women in Taiwan over a Lactation Period of One Year. *Biol Trace Elem Res* **1998**, *62*, 31–41, doi:10.1007/BF02820019.
57. Qian, J.; Chen, T.; Lu, W.; Wu, S.; Zhu, J. Breast Milk Macro- and Micronutrient Composition in Lactating Mothers from Suburban and Urban Shanghai: Breast Milk Analysis in Shanghai Women. *Journal of Paediatrics and Child Health* **2010**, *46*, 115–120, doi:10.1111/j.1440-1754.2009.01648.x.

58. Su, M.; Jia, H.; Chen, W.; Qi, X.; Liu, C.; Liu, Z. Macronutrient and Micronutrient Composition of Breast Milk from Women of Different Ages and Dietary Habits in Shanghai Area. *International Dairy Journal* **2018**, *85*, 27–34, doi:10.1016/j.idairyj.2018.04.015.
59. Wang, Y.; Zhang, Z.; Ge, P.; Wang, Y.; Wang, S. Iodine Status and Thyroid Function of Pregnant, Lactating Women and Infants (0-1 Yr) Residing in Areas with an Effective Universal Salt Iodization Program. *Asia Pacific Journal of Clinical Nutrition* **2009**, *18*, 34–40.
60. Zhang, M.; Chen, W.; Shen, J.; Zhao, Z.; Huo, J.; Lan, S.; Zhang, W. Iodine Concentrations in Breast Milk and Urine in Mothers and Infants During the Period of 16 Weeks Postpartum. *Acta Nutrimenta Sinica* **2014**, *36*, 548-552+627, doi:10.13325/j.cnki.acta.nutr.sin.2014.06.005.
61. Ren, X. The levels of B-vitamins in human milk and the effect factors[D], Chinese Center for Disease Control and Prevention, 2015. (in Chinese)
62. Ren, X.N.; Yin, S.; Yang, Z.; Yang, X.; Shao, B.; Ren, Y.; Zhang, J. Application of UPLC-MS/MS Method for Analyzing B-Vitamins in Human Milk. *Biomedical and Environmental Sciences 28*, 738–750, doi:10.3967/bes2015.104.
63. Ren, X.; Yang, Z.; Shao, B.; Yin, S.; Yang, X. B-Vitamin Levels in Human Milk among Different Lactation Stages and Areas in China. *PLoS ONE* **2015**, *10*, e0133285, doi:10.1371/journal.pone.0133285.
64. Xue, Y.; Redeuil, K.M.; Giménez, E.C.; Vinyes-Pares, G.; Zhao, A.; He, T.; Yang, X.; Zheng, Y.; Zhang, Y.; Wang, P.; et al. Regional, Socioeconomic, and Dietary Factors Influencing B-Vitamins in Human Milk of Urban Chinese Lactating Women at Different Lactation Stages. *BMC Nutr* **2017**, *3*, 22, doi:10.1186/s40795-017-0139-1.
65. Xue, Y.; Campos-Giménez, E.; Redeuil, K.; Lévèques, A.; Actis-Goretta, L.; Vinyes-Pares, G.; Zhang, Y.; Wang, P.; Thakkar, S. Concentrations of Carotenoids and Tocopherols in Breast Milk from Urban Chinese Mothers and Their Associations with Maternal Characteristics: A Cross-Sectional Study. *Nutrients* **2017**, *9*, 1229, doi:10.3390/nu9111229.
66. Sun, H.; Mao, Y.; Yang, X.; Cai, X.; Zhao, Y.; Chen, J.; Zhang, L. Carotenoids contents in breast milk from six areas of China. *Acta Nutrimenta Sinica* **2019**, *41*, 534–538, doi:10.13325/j.cnki.acta.nutr.sin.2019.06.003. (in Chinese)
67. Liu, J. Study on the Vitamin Contents of Human Milk in Huhhot. *Food Research and Development* **2016**, *37*, 20–22. (in Chinese)
68. Fang, F.; Li, T.; Li, Y.; Liu, B.; Ye, W. Investigation of the Contents of the Fat-Soluble Vitamins A, D and E in Human Milk from Hohhot. *Journal of Dairy Science and Technology* **2014**, *37*, 5–7, doi:10.15922/j.cnki.jdst.2014.03.013. (in Chinese)
69. Gao, S.; Zhou, X.; Chen, D. Analysis of Vitamin B12 and Folic Acid in Serum and Breast Milk during Late Pregnancy. *The Journal of Practical Medicine* **1994**, 316–317. (in Chinese)
70. Fang, F.; Li, T.; Liu, B.; Ye, W.; Yun, Z. Investigation of water-soluble vitamins B1, B2, B3 content in human milk from Hohhot. *China Dairy Industry* **2014**, *42*, 21–23. (in Chinese)

71. Zhang, L.; Bao, J.; Chen, H. Analysis of Vitamin A Determination Results in Breast Milk from Zhoushan Island. *Journal of Hygiene Research* **2001**, 234–236. (in Chinese)
72. Wu, K.; Sun, H.; Mao, Y.; Tian, F.; Cai, X.; Zhao, Y.; Cai, M. Natural RRR-alpha-tocopherol and synthetic alpha-tocopherol stereosiomers in human breast milk. *Acta Nutrimenta Sinica* **2019**, *41*, 539–543, doi:10.13325/j.cnki.acta.nutr.sin.2019.06.004. (in Chinese)
73. Zheng, M.; Quan, Y.; Cheng, Z.; Wang, Y.; Zhang, G. A Study on the Content and Correlation of Vitamin E in Maternal Blood, Umbilical Cord Blood, and Breast Milk. *Chinese Journal of Contemporary Pediatrics* **2001**, 305–306. (in Chinese)
74. Jiang, J.; Xiao, H.; Wu, K.; Yu, Z.; Ren, Y.; Zhao, Y.; Li, K.; Li, J.; Li, D. Retinol and α-Tocopherol in Human Milk and Their Relationship with Dietary Intake during Lactation. *Food Funct.* **2016**, *7*, 1985–1991, doi:10.1039/C5FO01293G.
75. Canfield, L.M.; Clandinin, M.T.; Davies, D.P.; Fernandez, M.C.; Jackson, J.; Hawkes, J.; Goldman, W.J.; Pramuk, K.; Reyes, H.; Sablan, B.; et al. Multinational Study of Major Breast Milk Carotenoids of Healthy Mothers. *Eur J Nutr* **2003**, *42*, 133–141, doi:10.1007/s00394-003-0403-9.
76. Zheng, M.C.; Zhou, L.S.; Zhang, G.F. Alpha-Tocopherol Content of Breast Milk in China. *J Nutr Sci Vitaminol (Tokyo)* **1993**, *39*, 517–520, doi:10.3177/jnsv.39.517.
77. Wu, K.; Zhu, J.; Zhou, L.; Shen, L.; Mao, Y.; Zhao, Y.; Gao, R.; Lou, Z.; Cai, M.; Wang, B. Lactational Changes of Fatty Acids and Fat-Soluble Antioxidants in Human Milk from Healthy Chinese Mothers. *Br J Nutr* **2020**, *123*, 841–848, doi:10.1017/S0007114520000239.
78. Wei, W.; Yang, J.; Xia, Y.; Chang, C.; Sun, C.; Yu, R.; Zhou, Q.; Qi, C.; Jin, Q.; Wang, X. Tocopherols in Human Milk: Change during Lactation, Stability during Frozen Storage, and Impact of Maternal Diet. *International Dairy Journal* **2018**, *84*, 1–5, doi:10.1016/j.idairyj.2018.03.009.
